# Effect of topical applications of sunflower seed oil on systemic fatty acid levels in under-two children under rehabilitation for severe acute malnutrition in Bangladesh: a randomized controlled trial

**DOI:** 10.1186/s12937-021-00707-3

**Published:** 2021-06-06

**Authors:** K. M. Shahunja, Daniel C. Sévin, Lindsay Kendall, Tahmeed Ahmed, Md. Iqbal Hossain, Mustafa Mahfuz, Xinyi Zhu, Krishan Singh, Sunita Singh, Jonathan M. Crowther, Rachel A. Gibson, Gary L. Darmstadt

**Affiliations:** 1 Nutrition and Clinical Services Division, International Centre for Diarrhoeal Disease Research, Bangladesh (icddr,b), Dhaka, Bangladesh; 2 Cellzome GmbH, GlaxoSmithKline R&D, Meyerhofstrasse 1, 69117 Heidelberg, Germany; 3 GlaxoSmithKline R&D, Gunnels Wood Road, Stevenage, Hertfordshire, UK; 4 JMC Scientific Consulting Ltd, Surrey, Egham UK; 5 Department of Pediatrics, Stanford University School of Medicine, 1701 Page Mill Road, Room 121, Palo Alto, Stanford, CA 94304 USA

**Keywords:** Topical application, Emollient, Sunflower seed oil, Fatty acids, Children, Malnutrition, Bangladesh

## Abstract

**Background:**

Children with severe acute malnutrition (SAM) have inadequate levels of fatty acids (FAs) and limited capacity for enteral nutritional rehabilitation. We hypothesized that topical high-linoleate sunflower seed oil (SSO) would be effective adjunctive treatment for children with SAM.

**Methods:**

This study tested a prespecified secondary endpoint of a randomized, controlled, unblinded clinical trial with 212 children with SAM aged 2 to 24 months in two strata (2 to < 6 months, 6 to 24 months in a 1:2 ratio) at Dhaka Hospital of icddr,b, Bangladesh between January 2016 and December 2017. All children received standard-of-care management of SAM. Children randomized to the emollient group also received whole-body applications of 3 g/kg SSO three times daily for 10 days. We applied difference-in-difference analysis and unsupervised clustering analysis using t-distributed stochastic neighbor embedding (t-SNE) to visualize changes in FA levels in blood from day 0 to day 10 of children with SAM treated with emollient compared to no-emollient.

**Results:**

Emollient therapy led to systematically higher increases in 26 of 29 FAs over time compared to the control. These effects were driven primarily by changes in younger subjects (27 of 29 FAs). Several FAs, especially those most abundant in SSO showed high-magnitude but non-significant incremental increases from day 0 to day 10 in the emollient group vs. the no-emollient group; for linoleic acid, a 237 μg/mL increase was attributable to enteral feeding and an incremental 98 *μg*/mL increase (41%) was due to emollient therapy. Behenic acid (22:0), gamma-linolenic acid (18:3n6), and eicosapentaenoic acid (20:5n3) were significantly increased in the younger age stratum; minimal changes were seen in the older children.

**Conclusions:**

SSO therapy for SAM augmented the impact of enteral feeding in increasing levels of several FAs in young children. Further research is warranted into optimizing this novel approach for nutritional rehabilitation of children with SAM, especially those < 6 months.

**Trial registration:**

ClinicalTrials.gov: NCT02616289.

**Supplementary Information:**

The online version contains supplementary material available at 10.1186/s12937-021-00707-3.

## Introduction

Severe acute malnutrition (SAM) affects an estimated 13.6 million children [1] – 300,000 in Bangladesh [2] – and causes about 500,000 deaths annually among under-five year-old children globally [2]. One-half to two-thirds of children with SAM present with diarrhea associated with enteropathy, maldigestion and malabsorption of dietary lipids, and increased intestinal permeability [3–8]. This leads to depletion of nutrients and useful gut microbiota, further resulting in deficiencies in energy harvest, vitamin biosynthesis and immune protection, and in increased risk of systemic invasion by microbial pathogens [9, 10].

Fatty acids (FAs) are integral to normal growth, health and development in childhood and are critical for cellular energy under starvation [11–13]. Levels of various FAs are low in tissues, plasma and erythrocytes of children with malnutrition [14, 15], and low levels of the adipose tissue hormone leptin are associated with higher child mortality [16]. While essential fatty acid (EFA) deficiency can be corrected through oral feeding of EFAs, provided that gut absorption is adequate, oral feeding may fail or be very slow in resolving signs of deficiency in the presence of compromised gut mucosal function in children with SAM [17]. Moreover, in Bangladesh, total consumption of fat is low among under-5 y-old children. An estimated 80% of children consume inadequate levels of linoleic acid: < 4% of total energy as compared with the recommended 4–8% [18].

The World Health Organization (WHO) has recommended ‘cautious feeding’ in the management of children with SAM with diarrhea and impaired gut physiology, recognizing their inability to tolerate usual amounts of dietary protein, fat and sodium [19]. It is recommended that therapeutic feeding start with a low-protein milk-based formula diet (F-75) during the stabilization or acute phase, followed by comparatively high protein and high calorie milk-based formula (F-100) in the rehabilitation phase. Ready-to-use therapeutic foods (RUTFs) – lipid-based pastes combining milk powder, electrolytes and micronutrients – have replaced F-100 in many settings for treatment of SAM in children older than age 6 months [20]. These oral feedings should be given according to management protocols to prevent refeeding syndrome and diarrhea, potentially leading to cardiac dysrhythmias and failure, respiratory distress and acute renal failure resulting in sudden death [20]. Although RUTF has a number of benefits including high caloric density and high levels of lipids and micronutrients, their cost is relatively high and limits their sustainability [21]. Some countries are developing RUTFs using locally available ingredients to reduce production costs [22, 23], or low-cost/high-calorie culturally acceptable cooked foods for SAM children in the rehabilitation phase [24].

Many developing countries where the prevalence of child malnutrition is high have tried to increase FA levels by supplementary feeding, especially with RUTFs, and have observed increases in some FAs levels by fortifying RUTFs with FAs [25], although poly-unsaturated FA (PUFA) requirements are not always met by current formulations [26]. Thus, RUTF as well as F-75 and F-100 formulas have limitations in their ability to correct EFA deficiency in children with SAM [21]. Given their gut dysfunction and the limitations in further augmenting enteral feeding regimens, new approaches are needed to increase FA levels in children with SAM.

Children with EFA deficiency, as seen in SAM, have disruptions in skin barrier function associated with increased losses of fluid and heat through the skin; increased energy utilization for biochemical homeostasis, immune defense, and skin repair; reduced growth, and increased risk of transcutaneous invasion of pathogens and mortality [27–30]. Topical emollient therapy, principally with sunflower seed oil (SSO) has been shown to augment skin barrier function leading to reduction in water and heat loss and preservation of energy [31, 32]. SSO is replete with EFAs and its major lipid constituents are PUFAs including high concentrations of linoleic acid [33]. In clinical trials in low resource settings, SSO therapy reduced risks for bloodstream infections and mortality and improved growth in very preterm, low birth weight infants [34–38]. Enhanced skin barrier function is central to the mechanism of action of emollient therapy in improving health of newborn infants [39–41]. Linoleic acid – the primary EFA component in SSO – binds specifically to receptors in keratinocytes which mediate skin development, thus accelerating this process [42–44], and has a direct role in epidermal barrier permeability repair, and repair of skin barrier function in states of nutritional deficiency [45–48].

Beyond local metabolic effects, FAs can be absorbed into the bloodstream following topical applications. This has been demonstrated in preterm and sick newborn infants as well as in adults with EFA deficiency who were unable to adequately ingest and absorb EFAs via the gut. Before the advent of intravenous lipids emulsions, these patients were treated successfully with high-linoleate topical SSO or safflower oil [32, 33, 49, 50]. Passive diffusion of oil occurs along a concentration gradient [51, 52], and can result in higher serum levels of various FAs in preterm neonates [50, 53].

We hypothesized that FAs in topical applications of SSO would improve skin barrier function locally and would also be absorbed, leading to systemic effects manifest in acceleration of clinical recovery of children with SAM beyond that achieved through standard-of-care oral feeding regimens. Previously we reported results from a clinical trial of SSO therapy in hospitalized young children 2 to 24 months of age with SAM in Bangladesh [54]. Here we examine the absorption of FAs into the bloodstream of the children with SAM in this trial who were treated with standard-of-care enteric feeding protocols plus topical applications of SSO compared to children treated with standard of care feeding protocols alone without use of emollients.

## Methods

### Study design and participants

This study tested a prespecified secondary endpoint of a randomized, two-arm, controlled, proof-of-concept clinical trial in Bangladesh to examine effects of emollient therapy as an adjunct intervention for children with SAM, as described previously [54]. Participants were recruited at Dhaka Hospital of the International Centre for Diarrhoeal Disease Research, Bangladesh (icddr,b), which annually treats more than 160,000 patients free-of-cost, the majority (59%) of whom are under 5 y old and from low-income families. The hospital has evidence-based management guidelines for children with SAM [24] and a state-of-the-art inpatient nutrition rehabilitation unit which annually treats about 1500 children under 5 y of age.

**Fig. 1:**
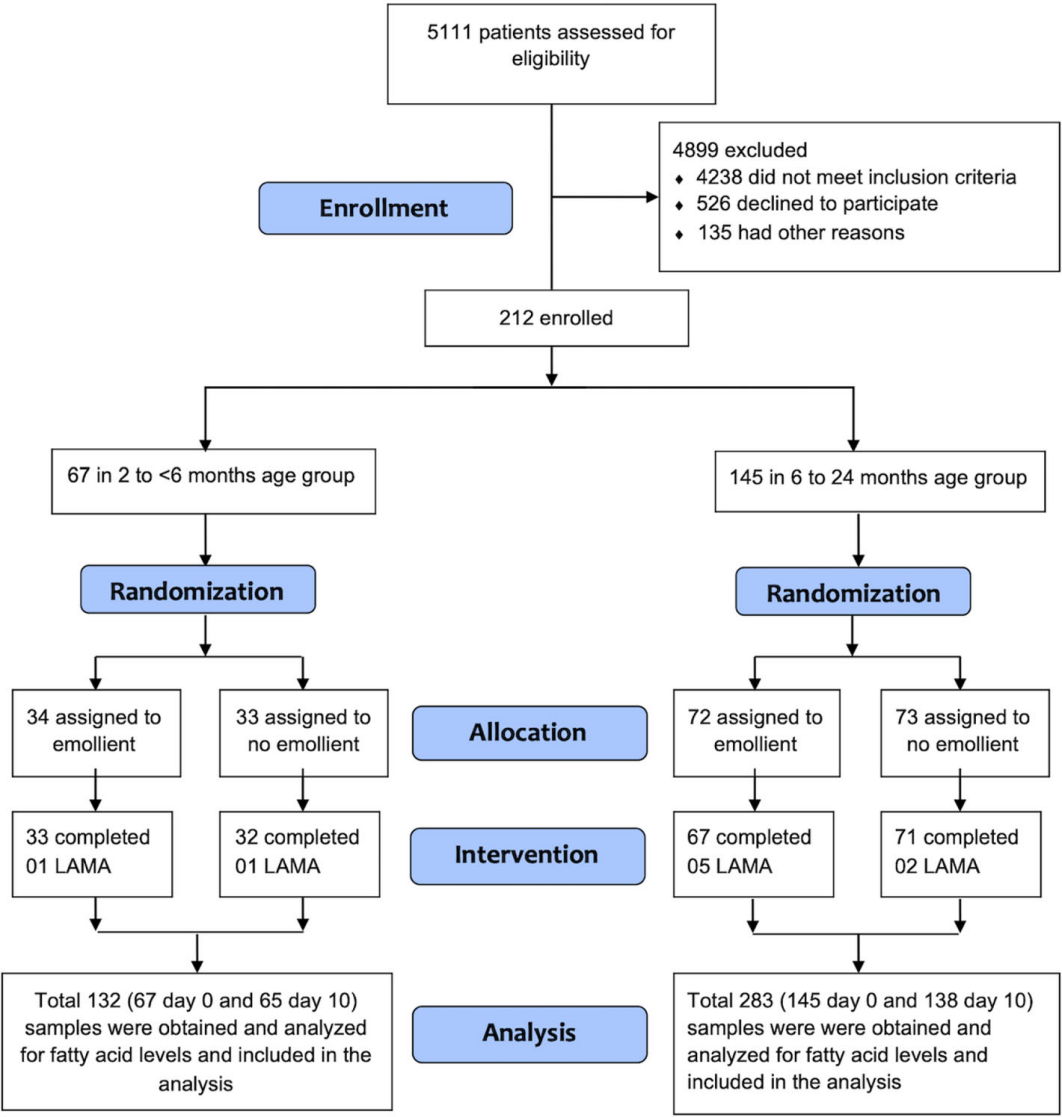
Trial profile

Boys and girls aged 2 to 24 months were prospectively enrolled after admission to the icddr,b hospital with a diagnosis of SAM [weight-for-length less than − 3 standard deviations (SDs) below the median for children under 5 y of age based on WHO growth standards [19], with complications such as diarrhea, pneumonia, etc., with or without nutritional edema] during January 2016 to December 2017 (Fig. 1). Participation was voluntary, and for all children written informed consent was obtained from the caregiver/legal guardian prior to enrollment. Other inclusion criteria included ability to comply with an inpatient stay of at least 10 days – the course of usual hospital treatment for SAM – and to suspend usual home skin care treatments for the same duration. Exclusion criteria included: the child was in custodial care (no longer looked after by their parent or legal guardian); the child presented with any life-threatening health condition such as septic shock and altered consciousness on admission, congenital disorders (e.g., congenital heart disease or known metabolic disorders, chromosomal abnormalities, etc.), any known chronic disease (e.g., tuberculosis, human immunodeficiency virus infection) or any known history of allergy to emollient therapy.

### Interventions

#### Standard-of-care for children with SAM

Standard-of-care treatment for children with SAM was divided into two stages, as described previously [24, 54]. Initial ‘acute phase’ treatment aimed to rescue the child from complications of their acute illnesses. This was followed by a ‘rehabilitation phase’ in which emphasis was placed on dietary measures to achieve catch-up growth. During acute phase management all the children received a milk-based liquid diet called ‘milk suji’(for children ages 6 months or more) or ‘modified infant formula’ (for non breast-fed children aged < 6 months). During the rehabilitation phase children > 6 months of age received two cooked foods called ‘Khicuri’ and ‘Halwa’ (Supplemental Table 1), consistent with emphasis on the use of locally sourced diets that can be prepared at home for treatment of children with SAM. No attempt was made to regulate food intake.

#### Topical emollient therapy

In addition to standard-of-care for SAM, participants who were randomized to the emollient therapy group were treated with 3 g of emollient per kg body weight per dose three times daily at 9 am, 5 pm and 11 pm – to integrate into usual patient care and minimize interruption of children’s and parents’ sleep – for 10 days. Emollient therapy consisted of cold pressed linoleic acid (18:2n6)-rich (48 to 74%), low oleic acid (18:1n9) (14–39%) containing SSO donated by a commercial supplier (Cargill Refined Oils, Europe) (Supplemental Table 2). SSO was stored at -20 °C in aliquots and defrosted to room temperature before use. The oil was maintained at room temperature for up to 7 days after which it was replaced with fresh oil. SSO was applied by dedicated study nurses trained in an appropriate method of gentle massage to ensure consistent application without injury to the skin. Oil was not applied to the face (to avoid the possibility of any accidental aspiration or ingestion), ears or scalp, or infected skin. The child’s anal area was washed prior to application to avoid the spread of fecal flora, and oil was applied there last. Bathing was delayed for a minimum of 3 to 4 h after application of emollient to optimize absorption of oil. The no-emollient (control) group did not receive any oil or massage.

### Outcome

Our prespecified outcome was changes in levels of serum fatty acids in blood from the time of enrollment to the end of 10 days of management in the hospital.

### Sample size

Given the lack of literature to enable us to preset specific endpoints around which we could power this study, we did not set out to detect a predetermined value of minimal important difference in a particular fatty acid or set of fatty acids; however, we aimed to explore whether there was a suggestion of differences in trends between the two study arms. Sample size for the main study was based on the expected rate of weight gain in the intervention (SSO) group compared to the no-emollient group, as described previously [54]. Based on that calculation, a minimum of 100 subjects per arm were required. Out of the total sample size of 200 participants pre-specified to be enrolled, two-thirds (*n* = 133) would be in the 6 to 24 months age stratum and one-third (*n* = 67) would be in the 2 to < 6 months age stratum. However, due to low enrollment of participants in the younger age stratum during the study period, we extended our enrollment period (after approval from the IRBs) in order to reach the original sample size estimate. During that time, we also continued enrollment in the older age stratum beyond our initial target sample size. Thus, the final number of participants was 212 young children.

### Randomization and concealment

Participants were randomized in a 1:1 ratio, with a permuted block size of four prepared in Microsoft Excel by a statistician who had no other role in this project – to receive either topical emollient therapy together with standard-of-care treatment for SAM or standard-of-care for SAM only for the 10-day duration of the study period (Fig. 1); details were described previously [54]. Randomization of participants was stratified in a 1:2 ratio by ages 2 to < 6 months and 6 to 24 months of age, respectively. It was not possible to mask study physicians and nurses to participant group assignment.

### Data collection

Socio-demographic, clinical history and examination, and laboratory data were collected from all participants by dedicated study physicians and research staff for the main trial [54]. Data on demography (age, sex), nutritional status (as z-score), randomization (to intervention or control), time points of intervention, daily breastfeeding practices, daily calorie intake from feeding other than breastfeeding (see Supplemental Table 3: 24-h Food Intake Chart), and daily rate of weight gain were used for this study as relevant. Daily caloric intake was determined by measuring the difference in weight of food offered minus leftover food and vomitus (if it happened immediately), for each meal, summing for all meals for the day. Weight of food was converted to caloric values using information in Supplemental Table 1.

Blood samples were drawn at the time of enrollment and after 10 days of hospital management. After collection of blood samples, serum was separated and stored at − 80 °C. Samples were shipped frozen to Metabolon (Morrisville, North Carolina, USA) for FA analysis using a proprietary method (TAM112) based on gas chromatography coupled to mass spectrometry (GC/MS). Briefly, after addition of internal standards, samples were dried under a stream of nitrogen and treated with methanol/sulfuric acid to convert free and conjugated FAs into FA methyl esters. Following neutralization and extraction with hexanes, an aliquot of the hexane layer was injected onto a 7890A/5975C GC/MS system (Agilent Technologies, Santa Clara, California) which was operated in single-ion monitoring positive mode with electron ionization. A panel of 29 FAs were determined, including seven saturated FAs [myristic acid (14:0), pentadecanoic acid (15:0), palmitic acid (16:0), stearic acid (18:0), arachidonic acid (20:0), behenic acid (22:0), and lignoceric acid (24:0)]; seven monounsaturated FAs [myristoleic acid (14:1n5), palmitoleic acid (16:1n7), vaccenic acid (18:1n7), oleic acid (18:1n9), cis-11-eicosaenoic acid (20:1n9), erucic acid (22:1n9), and nervonic acid (24:1n9)]; and 15 polyunsaturated FAs [mead acid (20:3n9), gamma-linolenic acid (18:3n6), dihomo-gamma-linolenic acid (20:3n6), arachidonic acid (20:4n6), adrenic acid (22:4n6), osbond acid (22:5n6), cis-11,14-eicosadienoic acid (20:2n6), cis-13-16-docosadienoic acid (22:2n6), alpha-linolenic acid (18:3n3), stearidonic acid (18:4n3), eicosatetraenoic acid (ETA) (20:4n3), EPA (20:5n3), docosapentaenoic acid (22:5n3), and DHA (22:6n3)]. Quantification was performed using both linear and quadratic calibration curves generated from authentic standards prepared immediately prior to each run.

### Statistical methods

Analysis of changes in fatty acid profiles from day 0 to day 10 for no emollient and emollient treated subjects was prespecified in the study’s reporting and analysis plan (Supplementary material for reference [54]. Data from FA analysis were reported as absolute concentrations in μg/mL. In some cases, FAs were not detected, which were resolved by reporting concentration values as “:” and excluding these values from statistical analyses. In other cases, FA levels were detectable but below the limit of quantitation (BLOQ) or above the limit of quantitation (ALOQ). In these cases, subject-specific extrapolated concentration values were reported and were used for statistical analyses. In some samples, levels of DHA (22:6n3) exceeded twice the concentration of the highest calibration standard. For these samples the quantitation software could not calculate an extrapolated concentration value above 320 μg/mL. However, to facilitate statistical analysis, the concentration in these samples was set at 320 μg/mL.

To quantify changes in FA levels in each of the 212 patients over time, FA concentrations at day 0 were subtracted from FA concentrations at day 10 for patients for whom data from both time points was available. Concentration differences over time in different patient groups were visualized by box-and-whisker plots, with patients grouped according to age category and treatment. Magnitude of concentration change differences over time between treatment groups was assessed by subtracting concentration changes in untreated patients from changes in emollient-treated patients [difference-in-difference (DID) analysis]. Statistical significance of concentration change differences over time between treatment groups was assessed by the nonparametric Wilcoxon test, with obtained *p*-values adjusted for multiple hypothesis testing using the Benjamini-Hochberg method [55].

To visualize the results of the DID analysis, log2-fold changes of each FA between day 10 and day 0 for untreated and emollient-treated patients were plotted against each other in scatter plots after separating patients by age group. Data were plotted as mean and standard deviation (in both x and y direction); statistically significant (Wilcoxon’s adjusted *p*-value < 0.05) fold-changes over time were highlighted.

t-distributed stochastic neighbor embedding (t-SNE) was used to project the dataset onto two dimensions by preserving distances between samples in the space of quantified FAs as much as possible [56]. Ultimately, this approach enables visualization of overall similarities between individual samples, and by overlaying patient metadata allows hypothesis testing on the contributions of different variables to underlying FA concentrations. t-SNE was performed on the full dataset (29 analytes, 415 samples), and after scanning a range of parameter values the final t-SNE parameter choices were to use the Manhattan distance metric and algorithm parameters k = 2, perplexity = 100 and max_iter = 1000. Factors examined included subject age group (2- < 6 months, 6–24 months) and sex (male, female), time point (day 0, day 10), treatment [emollient group, no-emollient group], breastfeeding status (exclusive, partial, none), rate of food intake (kcal/kg/d), and anthropometric measures [rate of weight gain (g/kg/d), weight-for-length z-score, length-for-age z-score]. t-SNE was analogously performed on the 212 data points resulting from the DID analysis. Subsequently data points (each representing a sample) were plotted as scatter plots with shapes and colors assigned according to metadata variable values.

### Trial oversight

An independent Data Safety and Monitoring Board (DSMB), formed by the IRB at icddr,b, provided oversight for the trial. All adverse events (AEs) and serious adverse events (SAEs) were recorded and reported through established processes at icddr,b. Unanticipated SAEs that were likely due to emollient application (such as the child slipping off the caregiver’s lap resulting in an injury, severe hypersensitivity reaction to the emollient, accidental choking or ingestion of emollient) were reported to the DSMB within 24 h of the event. AEs including nosocomial infection, sepsis, septic shock or any other anticipated consequence of the ongoing illness were not reported to the DSMB as per the instruction of the IRB, although all AEs and SAEs were recorded on the case record form of each participant.

## Results

### Study sample

Between January 2016 to December 2017, 5111 individuals were assessed for eligibility (Fig. 1). Among them, a total of 212 participants, 67 in the 2 to < 6 months age stratum and 145 in the 6 to 24 months age stratum, were enrolled. In the 6 to 24 months age stratum, 72 participants were enrolled in the emollient group and five participants left the hospital against medical advice before completion of the 10-day study period. Seventy-three participants were enrolled in the no-emollient (standard of care, no emollient therapy) group and 71 completed the 10-day study. In the 2 to < 6 months age stratum, 34 and 33 participants were enrolled in the emollient and no-emollient groups, respectively, and among them, 33 and 32 subjects completed the study. No participant died or developed any SAE during the study.

From 212 total participants, 415 blood samples were obtained for FA analysis; 203 participants – 65 children in the 2 to < 6 months age stratum and 138 children in the 6 to 24 months age stratum – had both baseline and endline samples for FA analysis (*n* = 406 samples) and nine subjects left the hospital during the study against medical advice and thus only contributed a baseline specimen (*n* = 9 samples).

Baseline demographic and clinical characteristics were comparable between the emollient and no-emollient groups in the two age strata, with the exception of the sex distribution of participants in the younger age stratum, in which more male participants were enrolled in the emollient than the no-emollient group (85% vs. 39%, respectively) (Table 1). Although there were some variations in types and amounts of diets among the participants, in both age strata the average calorie intakes from all sources of feeding except breastfeeding were comparable between emollient and no-emollient groups (Table 2). The median calorie intake in the younger age stratum was about 86 Kcal/kg body weight/day in the emollient group and 80 Kcal/kg body weight/day in the no-emollient group. In the older age stratum the average calorie intake was higher with a median of 98 Kcal/kg body weight/day in the emollient group and 100 Kcal/kg body weight/day in the no-emollient group. Average breastfeeding frequencies (per day) were also similar between the emollient and no-emollient groups in the two different age strata.

**Table 1:** Baseline characteristics of children with severe acute malnutrition enrolled in the trial of topical emollient therapy with sunflower seed oil vs control, by age strata (*n* = 212)

Variables	2 to < 6 months		6 to 24 months	
	Emollient (*n* = 34)	No emollient (*n* = 33)	Emollient (*n* = 72)	No emollient (*n* = 73)
Age, months (standard deviation, SD	4.07 (1.14)	4.05 (1.28)	11.97 (4.12)	12.81 (4.33)
Male sex, *n* (%)	29 (85%)	13 (39%)	49 (68%)	48 (66%)
Weight, kg (SD)	4.53 (0.87)	4.17 (0.67)	6.02 (0.80)	6.34 (0.85)
Length, cm (SD)	60.22 (4.04)	58.67 (3.10)	68.73 (4.55)	70.53 (4.78)
Weight-for-length z-score (SD)	−3.59 (0.40)	−3.46 (0.44)	− 3.52 (0.38)	−3.50 (0.49)
Mid-upper arm circumference, mm (SD)	112.82 (10.72)	109.58 (7.24)	119.75 (7.32)	122.77 (7.47)
Presence of oedema, *n* (%)	3 (9%)	1 (3%)	2 (3%)	1 (1%)
Duration of exclusive breastfeeding, months (SD)	1.03 (1.30)	0.56 (0.73)	2.28 (2.45)	2.73 (2.30)
Duration of diarrhea, days (SD)	4.41 (2.35)	4.33 (1.71)	4.25 (2.54)	4.58 (2.34)
Duration of fever, days (SD)	1.32 (2.29)	0.85 (1.00)	1.33 (2.15)	1.00 (1.20)
Haemoglobin, gm/dL (SD)	10.31 (1.29)	10.58 (2.47)	10.23 (1.81)	10.68 (1.66)

Data are *n* (%), mean (SD)

Adapted from Shahunja KM, et al. [54]

**Table 2:** Breastfeeding frequency and calorie intake (from all sources of feeding except breastfeeding) of children with severe acute malnutrition in the emollient vs. no-emollient groups, by age strata (*n* = 212)

Variables	2 to < 6 months		p*	6 to 24 months		p*
	Emollient (*n* = 34)	No emollient (*n* = 33)		Emollient (*n* = 72)	No emollient (*n* = 73)	
Breastfeeding frequency (per day) (median, IQR)	5.60 (0.00, 10.10)	7.65 (0.00, 10.45)	0.478	7.90 (0.00, 9.20)	8.60 (5.30, 9.80)	0.153
Calorie intake (per kg body wt. per day) (median, IQR)	86.50 (77.60, 93.60)	80.40 (72.00, 86.70)	0.088	98.00 (82.10, 123.00)	100.10 (81.80, 115.80)	0.975

*Mann Whitney *U* test

### Fatty acid levels

#### Quality control

Detectable ranges for each of the 29 FAs are shown in Supplemental Table 4. FA levels which were undetectable (Supplemental Table 5), BLOQ (Supplemental Table 6), ALOQ (Supplemental Table 7) and thus out-of-range are summarized in Supplemental Table 8. Instances of out-of-range were dominated by samples with undetectable levels of four FAs: cis-13-16-docosadienoic acid (22:2n6) in 92% of samples, (*n* = 381), stearidonic acid (18:4n3) in 75% (*n* = 312), erucic acid (22:1n9) in 55% (*n* = 229) and eicosatetraenoic acid (20:4n3) in 50% (*n* = 207). In general, undetectable samples were evenly distributed across day 0 and day 10 time points, the 6 to 24 month-old age group, and the treatment groups, but overall were less frequent in the 2 to < 6-month age group (Supplemental Table 5). No consistent pattern in levels BLOQ was seen across subgroups by time point, age group or treatment group (Supplemental Table 6). The frequency of samples ALOQ tended to be higher at either day 0 or at day 10, depending on the FA and tended to higher in the 6 to 24-month age group; there was no apparent difference by treatment group (Supplemental Table 7).

#### Treatment effects

**Fig. 2:**
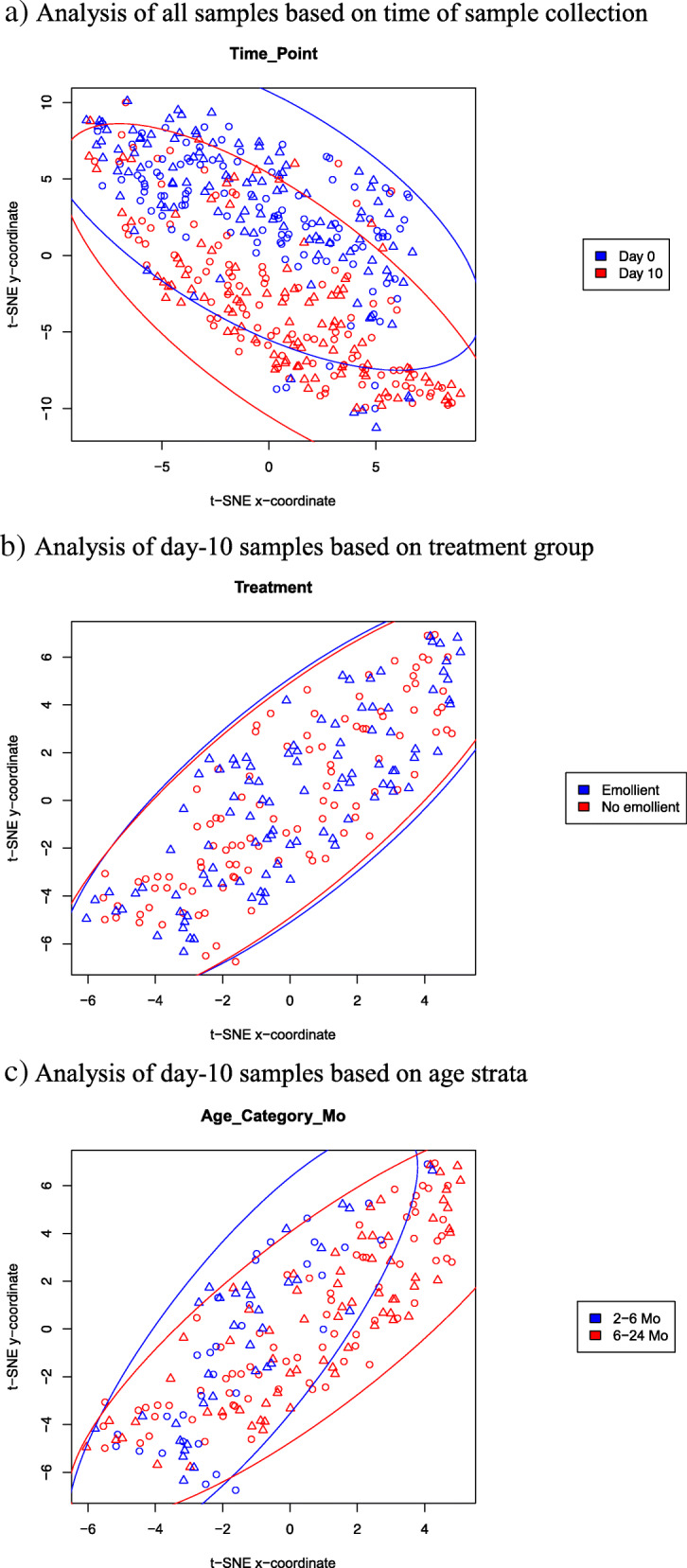
t-distributed stochastic neighbor embedding (t-SNE) performed on the full dataset of absolute fatty acid concentrations (29 analytes, 415 samples), plotting data points (each representing a sample) as scatter plots colored according to metadata variable values shown. Triangles indicate samples from emollient-treated and circles from no-emollient patients. **a**) Analysis of all samples based on time of sample collection. **b**) Analysis of day-10 samples based on treatment group. **c**) Analysis of day-10 samples based on age strata

**Fig. 3:**
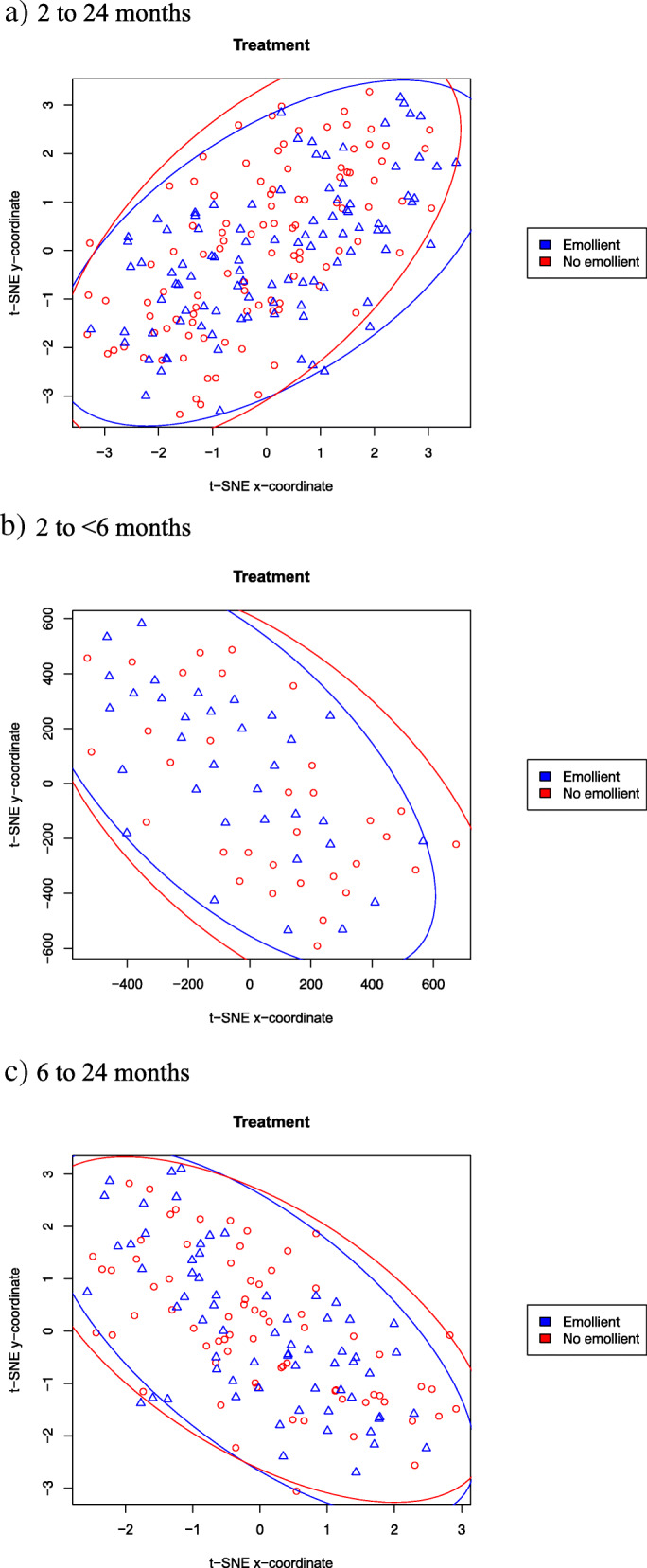
t-distributed stochastic neighbor embedding (t-SNE) difference-in-difference analysis performed between day-10 and day-0 samples of each patient, plotting data points (each representing a patient) as scatter plots colored according to treatment group, and shown for patients ages **a**) 2 to 24 months, **b**) 2 to < 6 months, and **c**) 6 to 24 months. Triangles indicate samples from emollient-treated and circles from no-emollient patients

Supplemental Table 9 shows the metadata that were used in analyzing changes in FA levels. Summary measures of fatty acid concentrations and variances are shown for emollient treated and no-emollient children with SAM in Supplemental Table 10. Analysis of absolute concentrations of FAs by t-SNE showed global segregation of samples based on time of collection on day 0 or day 10 (Fig. 2A). Further analysis showed no segregation by treatment group, including after 10 days of treatment (Fig. 2B), but revealed differences in the day-10 samples by age group (Fig. 2C). To examine whether the later result could be masking a more subtle treatment effect, we conducted t-SNE DID analysis which showed no separation by treatment group overall (Fig. 3A) or for either the younger (Fig. 3B) or the older age strata (Fig. 3C). Other variables examined – including gender, breastfeeding status, rate of weight gain, level of food intake, and anthropometric status (weight-for-length z-score, length-for-age z-score) – showed no segregation according to their absolute FA concentrations (Supplemental Fig. 1). Thus, t-SNE analysis revealed that among tested variables, time point had the strongest effect on global FA profiles. Additional methods were required to identify treatment effects that were more subtle and differences in individual FAs.

**Fig. 4:**
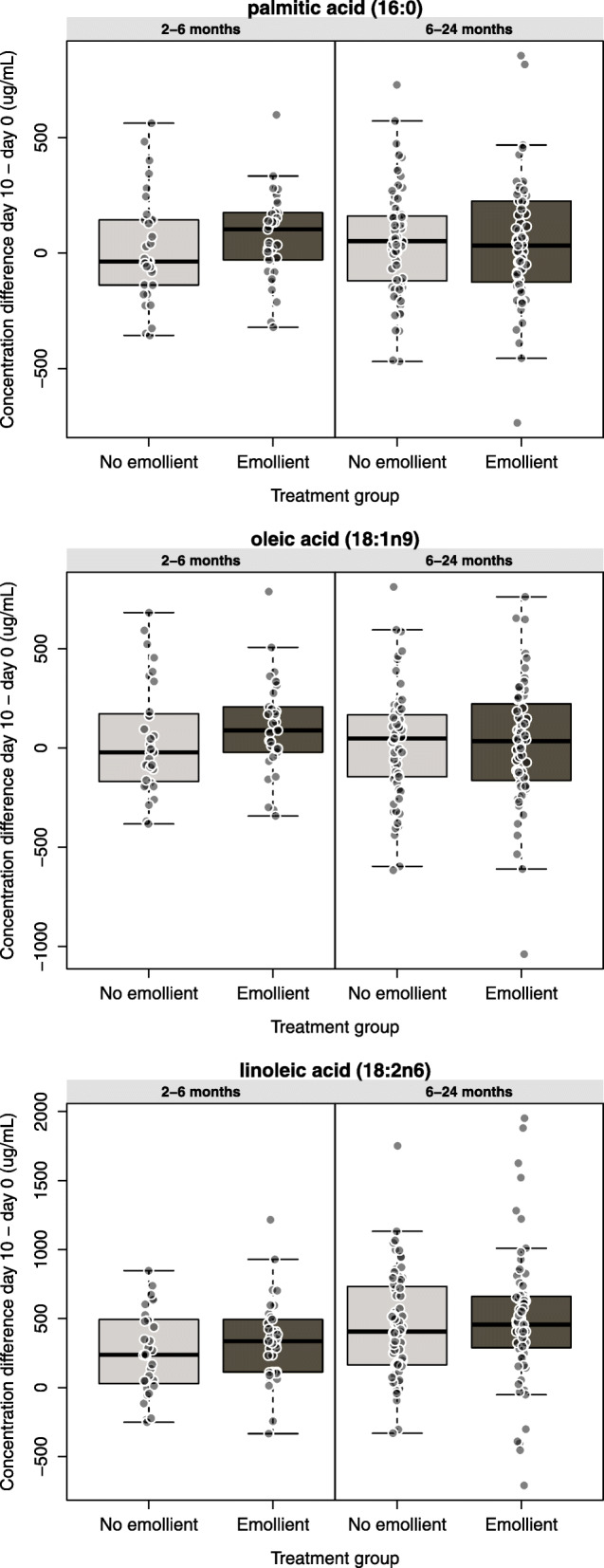
Box-and-whisker plots showing fatty acid concentration differences for **a**) palmitic acid (16:0), **b**) oleic acid (18:1n9), and **c**) linoleic acid (18:2n6) by treatment group and age strata. Individual data points represent the difference from day 0 to day 10 for the indicated fatty acid for an individual subject. Box-and-whisker plots represent data as mean (heavy horizontal line), lower and upper quartile (box edges), most extreme data points below 1.5 inter-quartile ranges from box edges (whiskers), and datapoints beyond 1.5 inter-quartile ranges from box edges (points beyond whiskers)

In DID analysis without adjusting for covariates, levels for most FAs were increased over the 10-day intervention period in both treatment groups and both age groups (Table 3, see columns showing Difference). In the younger age group, a few FAs – behenic acid (22:0), gamma-linolenic acid (18:3n6), and EPA (20:5n3) – showed statistically significant DID increases in the emollient group; the magnitudes of the increments were small but the proportional increases were large. When multiple testing was taken into account, all q-values were non-significant. No significant increases (DIDs) were seen in any FAs between day 0 and day 10 in the 6 to 24-month age group. In both age strata, the incremental increase from day 0 to day 10 (Difference) was notably highest for linoleic acid (18:2n6), more so in the emollient group than the no-emollient group, but the DIDs were not significant. In the younger age stratum (2 to < 6 months), DID values were highest for palmitic acid (16:0) (139.5 *μg*/mL), oleic acid (18:1n9) (110.6 *μg*/mL), and linoleic acid (18:2n6) (98.3 *μg*/mL) but were not significant (Fig. 4A-C). However, for linoleic acid,, the incremental 98 *μg*/mL increase due to emollient therapy represented a 41% gain over the 237 *μg*/mL increase attributable to enteral feeding. In the older age stratum (6 to 24 months), DID values overall were smaller than in the younger subjects, with the largest increase seen for linoleic acid (18:2n6) (51.2 *μg*/mL), but none were significant. Some FA level differences from day 0 to day 10 [especially palmitoleic acid (16:1n7), vaccenic acid (18:1n7), mead acid (20:3n9), arachidonic acid (20:4n6) and DHA (22:6n3)] were reduced in both age strata and both treatment groups.

**Table 3:** Difference (day 10 - day 0) and difference-in-difference (DID) in fatty acids levels in the blood of children with severe acute malnutrition in the emollient vs. the control group, by age strata (*n* = 203 children with both day 10 and day 0 samples, *n* = 406 samples included in fatty acid analysis)

Fatty acids (*μg*/mL)^**1**^	2 to < 6 months group					6 to 24 months group				
	Difference (day 10 minus day 0), Emollient *n* = 33 (Median)	Difference (day 10 minus day 0), No emollient *n* = 32 (Median)	DID (Emollient minus No emollient)	*p*-value^2^	q-value^3^	Difference (day 10 minus day 0), Emollient *n* = 67 (Median)	Difference (day 10 minus day 0), No emollient *n* = 71 (Median)	DID (Emollient minus No emollient)	*p*-value^2^	q-value^3^
myristic acid (14:0)	48.95	30.78	18.17	0.184	0.300	33.89	34.12	−0.23	0.661	0.983
pentadecanoic acid (15:0)	5.51	3.95	1.56	0.299	0.381	3.43	3.87	− 0.44	0.591	0.983
palmitic acid (16:0)	102.87	−36.59	139.46	0.171	0.300	32.24	51.65	−19.41	0.782	0.983
stearic acid (18:0)	50.12	25.33	24.78	0.193	0.300	46.76	61.64	− 14.89	0.493	0.983
arachidic acid (20:0)	0.85	0.52	0.33	0.318	0.387	0.88	0.86	0.02	0.939	0.983
behenic acid (22:0)	0.51	0.12	0.39	0.049**	0.294	0.68	0.74	−0.06	0.730	0.983
lignoceric acid (24:0)	0.23	− 0.07	0.30	0.087*	0.297	0.11	0.11	0.01	0.757	0.983
myristoleic acid (14:1n5)	2.38	0.88	1.49	0.078*	0.297	1.09	1.47	−0.38	0.583	0.983
palmitoleic acid (16:1n7)	−10.91	−26.67	15.75	0.063*	0.294	−23.01	−18.77	−4.24	0.775	0.983
vaccenic acid (18:1n7)	−3.68	−11.19	7.51	0.108	0.297	−6.22	−4.55	−1.66	0.966	0.983
oleic acid (18:1n9)	88.68	− 21.91	110.59	0.155	0.297	33.98	47.99	−14.01	0.983	0.983
cis-11-eicosaenoic acid (20:1n9)	0.70	−0.35	1.06	0.662	0.713	0.40	0.32	0.08	0.759	0.983
mead acid (20:3n9)	−0.95	−1.54	0.59	0.097*	0.297	−0.91	−0.86	− 0.05	0.900	0.983
erucic acid (22:1n9)	0.07	−0.18	0.25	0.129	0.297	0.20	−0.07	0.27	0.138	0.983
nervonic acid (24:1n9)	−0.58	−1.00	0.42	0.148	0.297	−1.60	−1.56	−0.05	0.875	0.983
linoleic acid (18:2n6)	335.77	237.44	98.33	0.270	0.377	456.43	405.21	51.22	0.399	0.983
gamma-linolenic acid (18:3n6)	2.92	0.86	2.06	0.038**	0.294	3.06	3.35	−0.28	0.761	0.983
cis-11,14-eicosadienoic acid (20:2n6)	3.15	2.96	0.20	0.502	0.585	4.68	4.38	0.30	0.597	0.983
dihomo-gamma-linolenic acid (20:3n6)	8.53	4.00	4.53	0.117	0.297	7.87	10.68	−2.81	0.307	0.983
arachidonic acid (20:4n6)	−22.11	−70.87	48.76	0.051*	0.294	−95.31	−81.92	−13.38	0.720	0.983
cis-13-16-docosadienoic acid (22:2n6)	−0.85	−0.37	−0.48	0.533	0.597	N/A	N/A	N/A	1.000	1.000
adrenic acid (22:4n6)	0.16	−0.14	0.30	0.053*	0.294	−.08	−0.07	−0.01	0.723	0.983
osbond acid (22:5n6)	0.07	−1.08	1.15	0.293	0.381	−2.25	−2.18	−0.08	0.892	0.983
alpha-linolenic acid (18:3n3)	11.53	10.78	0.75	0.809	0.839	25.52	20.47	5.05	0.389	0.983
stearidonic acid (18:4n3)	0.05	0.09	−0.04	1.000	0.880	0.92	N/A	N/A	1.000	1.000
eicosatetraenoic acid (20:4n3)	0.28	0.23	0.04	0.880	0.880	0.31	0.37	−0.06	0.742	0.983
eicosapentaenoic acid (20:5n3)	0.83	−2.12	2.96	0.023**	0.294	−1.84	−1.93	0.10	0.759	0.983
docosapentaenoic acid (22:5n3)	1.01	0.04	0.97	0.212	0.312	−1.16	−1.58	0.42	0.875	0.983
docosahexaenoic acid (22:6n3)	−3.61	−24.89	21.29	0.159	0.297	−44.91	−50.73	5.82	0.517	0.983

^1^See Supplemental Table 10 for summary day 0 and day 10 FA concentrations and measures of variance

^2^Mann–Whitney *U* test, ** = *p* < 0.05, * = *P* < 0.1

^3^*p*-values adjusted for multiple hypothesis testing using the Benjamini Hochberg false discovery rate controlling procedure [55]

**Fig. 5:**
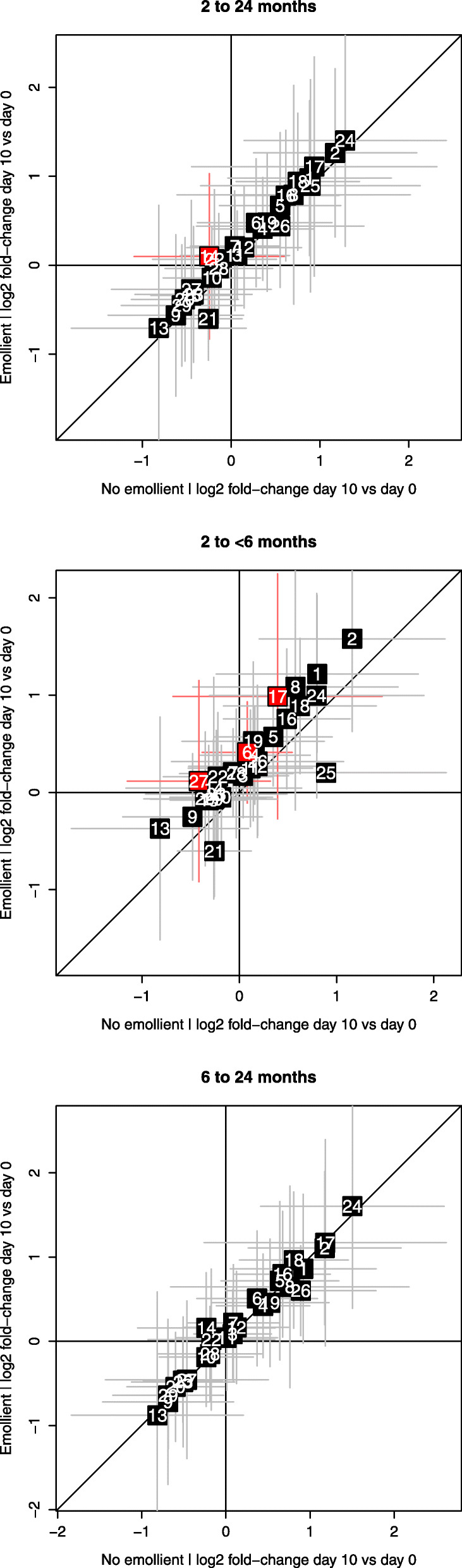
Mean log2 fold changes in fatty acids levels between day 10 and day 0 in emollient treated vs. not-emollient-treated patients in the following age groups: **a**) 2–24 months, **b**) 2- < 6 months, and **c**) 6–24 months. Fatty acids are identified by numbers referenced in Supplemental Table 10. Fatty acids with a raw *p*-value < 0.05 in the difference-in-difference analysis (Table 3) are highlighted in red

Plots of log2 fold-changes between day 10 and day 0 in patients treated with emollient vs not-emollient-treated patients (based on DIDs) showed that emollient therapy led to systematically higher increases in 26 of 29 FA levels over time compared to the control (i.e. the vertical off-set of most data points in the plot) (Fig. 5A). These effects were driven primarily by changes in the younger age group (27 of 29 FAs), in whom statistically significant increases were seen in behenic acid (22:0), gamma-linolenic acid (18:3n6), and EPA (20:5n3) (Fig. 5B). Minimal to no increases due to treatment were seen in the older age group (Fig. 5C).

## Discussion

Children with SAM have poorly functioning, highly permeable gut and skin barriers. Early and appropriate treatment for SAM is essential to prevent progression to the stage of severe complications that can often be fatal, yet limitations in the capacity of the diseased gastrointestinal system in children with SAM to absorb nutrients enterally is a key factor impeding rehabilitation. We hypothesized that the permeability of the skin barrier can be exploited for the topical delivery of essential nutrition in the form of FAs to accelerate clinical recovery. This is the first randomized controlled trial to examine the impact of topical applications of EFA-rich oil as an adjunctive treatment for nutritional rehabilitation of young children with SAM. Here we show that topical applications of SSO significantly increased the levels of several FAs in severely malnourished children in Bangladesh, particularly in the younger children aged 2 to < 6-months of age. This builds on a previous report in which we showed that a brief, 10-day period (the usual time period for hospitalization for rehabilitation of children with SAM) of topical therapy with SSO resulted in improved skin condition and skin barrier function in children 2 to 24 months of age, and reduction in nosocomial infections (primarily fever) and inflammatory markers (eg, C-reactive protein) in subgroups (by age) of treated children [54]. While treatment with emollient therapy showed a trend toward increased weight gain, especially in the younger patients (2 to < 6-months old), this effect did not reach statistical significance and time to clinical recovery from the acute phase of illness was not measurably impacted. Nevertheless, the clinical improvements seen within 10 days of topical therapy with SSO suggested that this modality has promise as an adjunctive treatment, warranting further research.

In this study, we found that the primary factor that enabled discrimination of FA profiles over the 10-day course of the study was time. The primary component of rehabilitation that these changes in FA levels can be attributed to over this period of time is the provision of nutrition – enteral and/or topical. The second most important factor associated with differences in FA profiles over the course of the study was patient age, with younger age associated with greater changes in FA profiles, consistent with the clinical observation of greater weight gain in the younger age group of patients [54]. FA profiles are known to change with maturation – with percentage contributions of linoleic acid (18:2n6) and alpha-linolenic acid (18:3n3) increasing and that from long-chain PUFAs decreasing after birth and into childhood [57] – which may explain in part the differential FA profiles we found by child age. Other factors, including patient sex, food or breastfeeding intake, or anthropometric factors (i.e., severity of malnutrition) were not associated with differences in FA profiles, consistent with prior studies [58, 59]. Given that emollient therapy did not emerge as a major factor in discriminating FA profiles suggests that enteral nutrition overall was the primary factor that influenced FA profiles. DID analysis of unadjusted levels of FAs corroborated this finding, given that the changes in individual FA levels from day 0 to day 10 of rehabilitation were largely similar in the no-emollient (enterally treated) and emollient (enterally plus topically treated) groups. However, the relatively large magnitudes (about 100–140 *μg*/mL) in the differences for linoleic acid (18:2n6), oleic acid (18:1n9), and palmitic acid (16:0) from day 0 to day 10 in the younger age group of emollient-treated vs no-emollient children, suggested that topical therapy may make important contributions to systemic levels of some FAs in children with SAM. However, the change in the linoleic acid (18:2n6) concentration in the blood of young control children (median 237 *μg*/mL), attributable to enteral feeding, exceeded the incremental additional change in the children also treated with emollient therapy (98 *μg*/mL), suggesting that enteral feeding was relatively more important in raising blood levels of this EFA. All children received the same types of therapeutic foods and all foods contained significant amounts of full cream milk powder and soybean oil, good sources of linoleic acid (18:2n6) [33]. Moreover, the average time for the children in our study to recover from acute illness was about 4 days, and when they recovered from acute illness their appetite improved and enteral caloric intake increased [54]. Nevertheless, the incremental 41% increase in linoleic acid (18:2n6) levels in the emollient group over the no-emollient group during the 10-day course of treatment suggests that cutaneous absorption made an important contribution. Moreover, in the younger patients (2 to < 6 months), small absolute but significant, large proportional increases were seen for three fatty acids [behenic acid (22:0), gamma-linolenic acid (18:3n6) and EPA (20:5n3)]. Large magnitude of cutaneous absorption also appeared to be important for palmitic acid (16:0) and oleic acid (18:1n9) – seemingly making a greater contribution than enteral absorption – as well as to a lesser extent for myristic acid (14:0) (where the contributions of cutaneous and enteral absorption appeared to be equal) and stearic acid (18:0) (for which cutaneous absorption appeared to be important but less so than for the enteral route). Importantly, these results mirror the FA composition of the SSO used for topical treatment. The FA composition of SSO was highest for linoleic acid (18:2n6) and oleic acid (18:1n9), and palmitic acid (16:0) and stearic acid (18:0) were also important but more minor components (Supplemental Table 2).

FA absorption through the skin occurs by diffusion, following Fick’s law [51]. Our results appear to reflect this principle and suggest that the FA composition of the oil is a key determinant in changes in FA profiles that may be seen in the blood following topical emollient therapy. We designed our intervention considering that emollients rich in omega-6 linoleic acid (18:2 n-6) are favored for skin barrier repair [31, 41, 60] and that the effect of emollient is likely to be most important early, in the first days of treatment when barrier disruption is maximal and a low-fat diet is being administered. SSO is replete with EFAs including linoleic acid (18:2n6) as its major lipid constituent [33]. Keratinocytes – the primary cell type comprising skin – have specific receptors that bind linoleic acid and facilitate its uptake [43]. Linoleic acid (18:2n6) converts to arachidonic acid (20:4n6) and is a precursor to prostaglandin E2, a known modulator of cutaneous inflammation [61]. Linoleate has also been shown to be among the most potent activators of peroxisome proliferator-activated receptor-α (PPARα), and its application to fetal rat skin explants at physiologic concentrations accelerated epidermal barrier development through stimulating PPARα and up-regulating genes controlling skin development [42, 62]. Linoleic acid also induces keratinocyte differentiation (i.e., expression of involucrin and transglutaminase) and inhibits proliferation of cultured keratinocytes, thus promoting skin maturation [42]. Although EFAs are not synthesized in situ, when applied exogenously their stimulation of PPARα and acceleration of barrier formation may account for some of the beneficial clinical effects seen with SSO application through enhancing skin barrier repair and function.

FA effects on the skin barrier must be balanced, however, with their effects following systemic absorption. Topical applications of SSO can accelerate barrier recovery over and above oral feeding in the context of compromise in fat absorption in the gut. In studies of single applications of radioactive SSO to the skin of EFA-deficient rats, only 10–20% of applied radioactivity was recovered in whole skin, and the majority was taken up by the skin, transported into the body and metabolized [63]. Studies in infants and adults have also shown absorption of lipids from the skin [32, 48, 49], although results are variable [64].

The impact of cutaneous applications of SSO beyond the skin in children with SAM is likely to be a dynamic process impacted by relative levels of cutaneous and gut absorption and metabolism of FAs, which may shift over the period of acute rehabilitation. Effects of cutaneous applications of SSO are seen rapidly (within a few days) and would be expected to result in reduced transepidermal water loss [as observed clinically [54]] and attenuation of transcutaneous absorption of fatty acids over the first week or so of treatment. Whereas the omega-6 FA, linoleic acid (18:2 n-6) is particularly beneficial for skin barrier formation and function, a diet rich in omega-3 fatty acids such as DHA (22:6n3) and EPA (20:5n3) is preferred for promotion of healthy neurodevelopment [65]. Furthermore, omega-6 FAs may block synthesis of omega-3 FAs. However, the few studies of the neurodevelopmental impact of treatment of preterm infants with oils high in omega-6 FAs over the course of the neonatal period have shown benefits [64, 66]. Moreover, foodstuffs in the dietary management of children with SAM – including the Milk Suzi, Khichuri and Halwa used in Bangladesh as well as RUTFs currently on the market – have about a 7:1 ratio of omega-6 to omega-3 FAs (n-6 FAs: 3–10%, n-3 FAs: 0.3–2.5%) [67]. Further research is warranted on the optimal mix of FAs for topical emollient treatment of SAM, keeping potentially differing requirements in mind for optimization of skin barrier and brain function and dynamic changes in rates of FA absorption as skin barrier function is repaired.

Our study had several limitations. We chose to measure plasma levels of FAs as levels in plasma reflect intake over weeks [68, 69], which was relevant to the time frame of our intervention study compared to changes in erythrocyte membrane FAs which reflect intake (and metabolism) over months. Given the short period of intervention in the study, and the lack of significant impact on weight gain [54], a longer trial of therapy which incorporates other measures of FA metabolism, such as erythrocyte membrane FAs, should be considered. Because the emollient intervention involved the application of oil with gentle massage, we could not separate the effects of massage from the transcutaneous absorption of oil. Massage in preterm infants has shown benefits in increased gastric activity and improved feeding tolerance and growth, raising the possibility that massage could improve enteral absorption of fatty acids derived from food and/or breastmilk [70–72]. Further research is needed to investigate this possibility.

Due to lack of healthy controls, we could not say how the levels of FAs in our children with SAM differ from their well-nourished peers. A study in Uganda showed that in SAM children, total and all n-6 PUFA including linoleic (18:2n6) and arachidonic acid (20:4n6) as well as total n-3 PUFA and DHA (22:6n3) were lower in children with SAM [58]. On the other hand, most monounsaturated FAs were found to be higher in children with SAM. In Burkina Faso, children with moderate acute malnutrition had low concentrations of whole-blood PUFAs, particularly n-3 PUFAs [14]. However, both these studies expressed FA values as weight percent of total FAs rather than absolute individual level in blood, so we could not compare the values of FAs in our children with these African children.

Although this study has shown that the topical application of high linoleate SSO increased the levels of some FAs, particularly those in highest abundance in the SSO, there were relatively few statistically significant differences. A possible reason for this is that the sample size of this study was not calculated based on FA endpoints, given the lack of data to inform the determination of these endpoints; rather, it was calculated based on the primary end point of the trial (i.e., rate of weight gain in g/kg/day). Findings from this study can help to inform the determination of predefined effect size and sample size for future research.

WHO global guidelines for management of SAM in children under 5 years of age [20] include infants < 6 months; however, more evidence is needed to inform guidelines for management of SAM in young infants. Management of SAM is yet to be scaled-up in countries where it is needed most, partly because of the complexity of the recommendations and lack of corresponding therapeutic essentials, resources and trained workers. Cost is also a major constraint to sustainability. Topical emollient therapy was shown to be highly cost effective and promising for management of hospitalized preterm infants in Bangladesh [73]. Further research on cost effectiveness in treatment of children with SAM is needed.

## Conclusion

Our results indicate that topical therapy with linoleic-acid rich SSO played a minor yet promising role in augmenting the impact of enteral feeding in increasing levels of some FAs in young children with SAM under rehabilitation in the hospital. Given the concordance between the FA composition of the oil and the FAs which showed the greatest magnitude of cutaneous absoorption, it may be possible to further improve topical products to optimize the absorption of the most beneficial FAs. Further research is warranted to explore the use of topical emollient therapy as an adjunctive treatment for nutritional rehabilitation of young children with SAM.

## Supplementary Information

**Additional file 1: Supplemental Tables and figures. Supplemental Table 1.** Composition of diets with calorie values for children with severe acute malnutrition (SAM) during rehabilitation. **Supplemental Table 1A**. Composition of liquid diets during acute phase of treatment of children with SAM. **Supplemental Table 1B**. Composition of semi-solid diets during rehabilitation phase of treatment for SAM. **Supplemental Table 2.** Specification of emollient (sunflower seed oil). **Supplemental Table 3.** 24-h Food Intake Chart. **Supplemental Table 4.** Lower and upper limits of detection provided by Metabolon for their free fatty acid analysis platform in *u*g/mL. **Supplemental Table 5.** Fatty acid measurements where an analyte was not detected. **Suppplemental Table 6.** Fatty acid measurements where an analyte was below the limit of quantitation (BLOQ). **Supplemental Table 7.** Fatty acid measurements where an analyte was above the limit of quantitation (ALOQ). **Supplemental Table 8.** Summary of out-of-range measurements of fatty acid levels. **Supplemental Table 9**. Metadata used for statistical analysis of fatty acid levels in children with severe acute malnutrition. **Supplemental Table 10.** Summary statistics on fatty acid concentrations. **Supplemental Figure 1**. t-distributed stochastic neighbor embedding (t-SNE) of absolute concentrations performed on samples from both study timepoints (i.e., day 0 and day 10), plotting data points (each representing a sample) as scatter plots shown for patients ages 2 to 24 months (left column), 2 to < 6 months (middle column), and 6 to 24 months (right column). Triangles indicate samples from emollient-treated and circles from no-emollient patients.

## Data Availability

Trial data are not publicly available owing to data privacy, but access to the anonymized dataset can be obtained from the corresponding author on reasonable request. The study Reporting and Analysis Plan is available at: https://www.jogh.org/documents/issue202001/jogh-10-010414-s001.pdf.

## Abbreviations

ALOQ: Above the limit of quantitation
AEs: Adverse events
BLOQ: Below the limit of quantitation
DSMB: Data Safety and Monitoring Board
DID: Difference-in-difference
DHA: Docosahexaenoic acid (22:6n3)
EPA: Eicosapentaenoic acid (20:5n3)
EFA: Essential fatty acid
FAs: Fatty acids
GC/MS: Gas chromatography coupled to mass spectrometry
IRB: Institutional Review Board
PPARα: Peroxisome proliferator-activated receptor-α
PUFA: Poly-unsaturated fatty acid
RUTF: Ready-to-use therapeutic food
SAEs: Serious adverse events
SAM: Severe acute malnutrition
SD: Standard deviation
SSO: Sunflower seed oil
t-SNE: T-distributed stochastic neighbor embedding
WHO: World Health Organization
YLL: Years of life lost
