# Supplementary material for: Effect of topical applications of sunflower seed oil on systemic fatty acid levels in under-two children under rehabilitation for severe acute malnutrition in Bangladesh: a randomized controlled trial
Source: Nutr J. 2021 Jun 6;20:51. doi: 10.1186/s12937-021-00707-3 (PMC8183055; doi:10.1186/s12937-021-00707-3)

**Supplemental Tables and Figures**

**Supplemental Table 1.** Composition of diets with calorie values for children with severe acute malnutrition (SAM) during rehabilitation

**Supplemental Table 1A**. Composition of liquid diets during acute phase of treatment of children with SAM

|  | **Infant formula** | **Milk suji** | **Milk suji 100** |
| --- | --- | --- | --- |
| Whole milk powder (g) | 60 | 40 | 80 |
| Rice powder (g) | − | 40 | 50 |
| White of eggs (g) | − | − | − |
| Sugar (g) | 50 | 25 | 50 |
| Soya oil (g) | 20 | 25 | 25 |
| Magnesium chloride (g) | 0.5 | 0.5 | 0.5 |
| Potassium chloride (g) | 1.0 | 1.0 | 1.0 |
| Calcium lactate (g) | 2.0 | 2.0 | 2.0 |
| Cooked volume (L) | 1.0 | 1.0 | 1.0 |
| Energy (kcal/100 mL) | 68 | 67 | 100 |
| *PER % | 9 | 8 | 10 |
| **FER % | 47 | 47 | 40 |

*****PER is protein-energy ratio, **FER is fat-energy ratio

**Supplemental Table 1B**. Composition of semi-solid diets during rehabilitation phase of treatment for SAM

| **Ingredient** | **Khichuri** | **Halwa** |
| --- | --- | --- |
| Rice | 120 g |  |
| Wheat flour (atta) | - | 200 g |
| Lentils (mashur dal) | 60 g | 100 g |
| Oil (soya) | 70 mL | 100 mL |
| Molasses (brown sugar) | - | 125 g |
| Potato | 100 g | - |
| Pumpkin | 100 g | - |
| Leafy vegetable (shak) | 80 g | - |
| Onion (2 medium size) | 50 g | - |
| Spices * | 50 g | - |
| Water | 1,000 mL | 600 mL |
| Total weight | 1,000 g | 1,000 g |
| Total energy per kg | 1,442 kcal | 2,404 kcal |

**Supplemental Table 2.** Specification of emollient (sunflower seed oil)

| ***TECHNICAL SPECIFICATIONS ^1^*** | | | | | |
| --- | --- | --- | --- | --- | --- |
|  |  | **Min** | | **Max** | ***Reference method ^2)^*** |
| **Sensory**: | | | | | |
| Taste | - | bland | |  | *Cargill Internal method* |
| Appearance at room temperature | - | clear | |  | *Cargill Internal method* |
| **Chemical**: | | | | | |
| Free Fatty Acid, as oleic | % | | - | 0.10 | *EN-ISO 660:2009* |
| Peroxide Value, at bottling | meq/kg | | - | 2.0 | *ISO 3960:2007* |
| Moisture Content | % | | - | 0.10 | *ISO 8534:2007* |
| Colour Lovibond 5.25” | Red | | - | 2.0 | *ISO 15305:1998* |
| **Fatty Acid Composition:** | | *EN-ISO 5509:2000 & EN-ISO 5508:1995* | | | |
| C16:0 (palmitic acid) | % | 5.0 | | 7.6 |  |
| C18:0 (stearic acid) | % | 2.7 | | 6.5 |  |
| C18:1 (total) (oleic acid) | % | 14.0 | | 39.4 |  |
| C18:2 (total) (linoleic acid) | % | 48.3 | | 74.0 |  |
| C18:3 (total) | % | - | | 0.5 |  |
| Trans fatty acids (total) | % | - | | 2.0 |  |

*1) Analyses are done by our refineries/ suppliers before reception of the oils in the bottling plant*

*2) Cargil (commercial supplier)l reserves the right to use internal analytical method that is in compliance with the International Reference Method*

**Supplemental Table 3.** 24-hour Food Intake Chart

**Topical emollient therapy in the management of SAM, icddr,b**

**Protocol Number: Participant’s ID: Daily food intake chart**

***Use one sheet per day***

**Treatment phase: Acute / Rehabilitation**

Hospital ID: **_/__/__/__/__/__/** Patient’s Name: ______________________ Age: **__/__/**

Date: **__/__/__/__/__/__/**  Today’s body weight (kg): **__/__/. __/__/__/**

| **Time** | **Type of food** | **Energy value of the food (kcal/gm)** | **Amount of food offered (gm)**  **(A)** | **Amount left (gm)**  **(B)** | **Estimated amount of vomiting (gm)**  **(C)** | **Amount eaten (excluding vomiting)**  **[D = A – (B+C)]** | **Total energy (kcal)** |
| --- | --- | --- | --- | --- | --- | --- | --- |
|  |  |  |  |  |  |  |  |
|  |  |  |  |  |  |  |  |
|  |  |  |  |  |  |  |  |
|  |  |  |  |  |  |  |  |
|  |  |  |  |  |  |  |  |
|  |  |  |  |  |  |  |  |
|  |  |  |  |  |  |  |  |
|  |  |  |  |  |  |  |  |
|  |  |  |  |  |  |  |  |
|  |  |  |  |  |  |  |  |
|  |  |  |  |  |  |  |  |
|  |  |  |  |  |  |  |  |
| **Total in Last 24 hours** |  |  |  |  |  |  |  |

Frequency of breast feeding in last 24 hours: **__/__/**

**Supplemental Table 4.** Lower and upper limits of detection provided by

Metabolon for their free fatty acid analysis platform in *u*g/mL

| **Analyte** | Lower limit of detection (*u*g/mL) | Upper limit of detection (*u*g/mL) |
| --- | --- | --- |
| myristic acid (14:0) | 1.130 | 151.000 |
| myristoleic acid (14:1n5) | 0.565 | 75.300 |
| pentadecanoic acid (15:0) | 0.570 | 76.000 |
| palmitic acid (16:0) | 7.410 | 988.000 |
| palmitoleic acid (16:1n7) | 1.170 | 157.000 |
| stearic acid (18:0) | 2.950 | 393.000 |
| oleic acid (18:1n9) | 8.950 | 1193.000 |
| vaccenic acid (18:1n7) | 0.600 | 80.000 |
| linoleic acid (18:2n6) | 8.980 | 1197.000 |
| gamma-linolenic acid (18:3n6) | 0.574 | 76.500 |
| alpha-linolenic acid (18:3n3) | 0.577 | 76.900 |
| stearidonic acid (18:4n3) | 0.600 | 80.000 |
| arachidic acid (20:0) | 1.150 | 154.000 |
| cis-11-eicosaenoic acid (20:1n9) | 0.577 | 76.900 |
| mead acid (20:3n9) | 0.600 | 80.000 |
| cis-11,14-eicosadienoic acid (20:2n6) | 0.578 | 77.100 |
| dihomo-gamma-linolenic acid (20:3n6) | 0.590 | 78.700 |
| arachidonic acid (20:4n6) | 4.180 | 558.000 |
| eicosatetraenoic acid (20:4n3) | 0.600 | 80.000 |
| eicosapentaenoic acid (20:5n3) | 0.595 | 79.400 |
| behenic acid (22:0) | 1.160 | 154.000 |
| erucic acid (22:1n9) | 1.160 | 77.200 |
| cis-13-16-docosadienoic acid (22:2n6) | 0.621 | 82.800 |
| adrenic acid (22:4n6) | 0.600 | 80.000 |
| osbond acid (22:5n6) | 0.600 | 80.000 |
| docosapentaenoic acid (22:5n3) | 0.600 | 80.000 |
| docosahexaenoic acid (22:6n3) | 1.200 | 160.000 |
| lignoceric acid (24:0) | 1.160 | 154.000 |
| nervonic acid (24:1n9) | 0.579 | 77.200 |

**Supplemental Table 5.** Fatty acid measurements where an analyte was not detected

| **biochemical** | **total # of samples in which measurement was not detected** | **of which day 0** | **of which day 10** | **of which 2-<6 m** | **of which 6-24 m** | **of which treated with emollient** | **of which no-emollient** |
| --- | --- | --- | --- | --- | --- | --- | --- |
| cis-13-16-docosadienoic acid (22:2n6) | 381 (92%) | 193 | 188 | 102 | 279 | 189 | 192 |
| stearidonic acid (18:4n3) | 312 (75%) | 197 | 115 | 96 | 216 | 161 | 151 |
| erucic acid (22:1n9) | 229 (55%) | 117 | 112 | 40 | 189 | 110 | 119 |
| eicosatetraenoic acid (20:4n3) | 207 (50%) | 142 | 65 | 47 | 160 | 100 | 107 |
| mead acid (20:3n9) | 3 (0.5%) | 0 | 3 | 0 | 3 | 2 | 1 |
| adrenic acid (22:4n6) | 3 (0.5%) | 2 | 1 | 0 | 3 | 2 | 1 |
| nervonic acid (24:1n9) | 1 (0.2%) | 0 | 1 | 0 | 1 | 0 | 1 |
| gamma-linolenic acid (18:3n6) | 1 (0.2%) | 1 | 0 | 0 | 1 | 1 | 0 |
| eicosapentaenoic acid (20:5n3) | 1 (0.2%) | 0 | 1 | 1 | 0 | 1 | 0 |

**Suppplemental Table 6.** Fatty acid measurements where an analyte was below the limit of quantitation (BLOQ)

| **biochemical** | **total # of samples in which measurement was BLOQ** | **of which day 0** | **of which day 10** | **of which 2-6 m** | **of which 6-24 m** | **of which treated with emollient** | **of which no-emollient** |
| --- | --- | --- | --- | --- | --- | --- | --- |
| erucic acid (22:1n9) | 130 (31%) | 65 | 65 | 81 | 49 | 60 | 70 |
| adrenic acid (22:4n6) | 66 (16%) | 32 | 34 | 7 | 59 | 39 | 27 |
| stearidonic acid (18:4n3) | 54 (13%) | 12 | 42 | 27 | 27 | 27 | 27 |
| eicosatetraenoic acid (20:4n3) | 43 (10%) | 17 | 26 | 3 | 40 | 19 | 24 |
| lignoceric acid (24:0) | 42 (10%) | 23 | 19 | 11 | 31 | 25 | 17 |
| arachidic acid (20:0) | 36 (9%) | 23 | 13 | 5 | 31 | 20 | 16 |
| mead acid (20:3n9) | 19 (5%) | 5 | 14 | 1 | 18 | 9 | 10 |
| behenic acid (22:0) | 18 (4%) | 12 | 6 | 5 | 13 | 12 | 6 |
| myristoleic acid (14:1n5) | 5 (1%) | 2 | 3 | 1 | 4 | 5 | 0 |
| gamma-linolenic acid (18:3n6) | 1 (0%) | 0 | 1 | 0 | 1 | 1 | 0 |

**Supplemental Table 7.** Fatty acid measurements where an analyte was above the limit of quantitation (ALOQ)

| **biochemical** | **total # of samples in which measurement was ALOQ** | **of which day 0** | **of which day 10** | **of which 2-<6 m** | **of which 6-24 m** | **of which treated with emollient** | **of which no-emollient** |
| --- | --- | --- | --- | --- | --- | --- | --- |
| docosahexaenoic acid (22:6n3) | 79 (19%) | 64 | 15 | 12 | 67 | 36 | 43 |
| linoleic acid (18:2n6) | 74 (18%) | 8 | 66 | 7 | 67 | 39 | 35 |
| vaccenic acid (18:1n7) | 56 (13%) | 39 | 17 | 32 | 24 | 26 | 30 |
| palmitic acid (16:0) | 30 (7%) | 8 | 22 | 12 | 18 | 17 | 13 |
| myristic acid (14:0) | 25 (6%) | 1 | 24 | 9 | 16 | 9 | 16 |
| alpha-linolenic acid (18:3n3) | 24 (6%) | 1 | 23 | 2 | 22 | 11 | 13 |
| palmitoleic acid (16:1n7) | 11 (3%) | 9 | 2 | 8 | 3 | 3 | 8 |
| oleic acid (18:1n9) | 10 (2%) | 2 | 8 | 4 | 6 | 6 | 4 |
| arachidonic acid (20:4n6) | 4 (1%) | 3 | 1 | 0 | 4 | 1 | 3 |
| stearic acid (18:0) | 3 (1%) | 0 | 3 | 2 | 1 | 2 | 1 |
| dihomo-gamma-linolenic acid (20:3n6) | 2 (0%) | 0 | 2 | 1 | 1 | 1 | 1 |

**Supplemental Table 8.** Summary of out-of-range measurements of fatty acid levels

| **biochemical** | **total # of samples in with out-of-range measurements of any type** | **total # of samples in which measurement was “not detected”** | **total # of samples in which measurement was BLOQ** | **total # of samples in which measurement was ALOQ** |
| --- | --- | --- | --- | --- |
| cis-13-16-docosadienoic acid (22:2n6) | 381 (92%) | 381 (92%) | - | - |
| stearidonic acid (18:4n3) | 366 (88%) | 312 (75%) | 54 (13%) | - |
| erucic acid (22:1n9) | 359 (87%) | 229 (55%) | 130 (31%) | - |
| eicosatetraenoic acid (20:4n3) | 250 (60%) | 207 (50%) | 43 (10%) | - |
| docosahexaenoic acid (22:6n3) | 79 (19%) | - | - | 79 (19%) |
| linoleic acid (18:2n6) | 74 (18%) | - | - | 74 (18%) |
| adrenic acid (22:4n6) | 69 (17%) | 3 (1%) | 66 (16%) | - |
| vaccenic acid (18:1n7) | 56 (13%) | - | - | 56 (13%) |
| lignoceric acid (24:0) | 42 (10%) | - | 42 (10%) | - |
| arachidic acid (20:0) | 36 (9%) | - | 36 (9%) | - |
| palmitic acid (16:0) | 30 (7%) | - | - | 30 (7%) |
| myristic acid (14:0) | 25 (6%) | - | - | 25 (6%) |
| alpha-linolenic acid (18:3n3) | 24 (6%) | - | - | 24 (6%) |
| mead acid (20:3n9) | 22 (5%) | 3 (1%) | 19 (5%) | - |
| behenic acid (22:0) | 18 (4%) | - | 18 (4%) | - |
| palmitoleic acid (16:1n7) | 11 (3%) | - | - | 11 (3%) |
| oleic acid (18:1n9) | 10 (2%) | - | - | 10 (2%) |
| myristoleic acid (14:1n5) | 5 (1%) | - | 5 (1%) | - |
| arachidonic acid (20:4n6) | 4 (1%) | - | - | 4 (1%) |
| stearic acid (18:0) | 3 (1%) | - | - | 3 (1%) |
| gamma-linolenic acid (18:3n6) | 2 (0%) | 1 (0%) | 1 (0%) | - |
| dihomo-gamma-linolenic acid (20:3n6) | 2 (0%) | - | - | 2 (0%) |
| nervonic acid (24:1n9) | 1 (0.2%) | 1 (0%) | - | - |
| eicosapentaenoic acid (20:5n3) | 1 (0.2%) | 1 (0%) | - | - |

BLOQ, below the limit of quantification; ALOQ, above the limit of quantification

**Supplemental Table 9**. Metadata used for statistical analysis of fatty acid levels in children with severe acute malnutrition

| **Variable name** | **Variable description** | **Data type** | **# Categories or range** | **Values (if categorical)** |
| --- | --- | --- | --- | --- |
| Treatment | topical treatment with emollient | categorical | 2 | Emollient, No emollient |
| Time_Point | time point of sample collection (days post beginning of treatment) | categorical | 2 | Day 0, Day 10 |
| Gender | subject gender | categorical | 2 | F, M |
| Age_Mo | subject age (months) | continuous | 2.0 – 22.7 months | - |
| Age_Category_Mo | subject age group (months) | categorical | 2 | 2 – <6 Mo, 6-24 Mo |
| Breastfeeding_Status | breast feeding status | categorical | 3 | Exclusive breast feeding, Partial breast feeding, No breast feeding since birth |
| Weight_Gain_Rate_gkgd | rate of weight gain after 11 days of study (in g gained weight / kg body weight / day) | continuous | -6.2 – 19.0 g/(kg*day) | - |
| Food_Intake_Rate_kcalkgd | rate of caloric intake after 11 days of study (in kcal / kg body weight / day) | continuous | 20.7 – 183.7 kcal/(kg*day) | - |
| Weight_For_Length_Zsc | subject weight/length ratio compared to reference population (Z-score, i.e. standard deviations from mean) | continuous | -5.4 – -1.0 | - |
| Length_For_Age_Zsc | subject length/age ratio compared to reference population (Z-score, i.e. standard deviations from mean) | continuous | -6.1 – 1.6 | - |

**Supplemental Table 10.** Summary statistics on fatty acid concentrations

|  |  | **Day 0** | | | | | | | | **Day 10** | | | | | | | |
| --- | --- | --- | --- | --- | --- | --- | --- | --- | --- | --- | --- | --- | --- | --- | --- | --- | --- |
|  |  | **Mean** | **Median** | **Standard deviation** | **Minimum** | **Maximum** | **25th percentile** | **75th percentile** | **Number of measurements** | **Mean** | **Median** | **Standard deviation** | **Minimum** | **Maximum** | **25th percentile** | **75th percentile** | **Number of measurements** |
| **Fatty acid name** | **Fatty acid index (label on scatter plots)** | (µg/mL) | (µg/mL) | (µg/mL) | (µg/mL) | (µg/mL) | (µg/mL) | (µg/mL) | - | (µg/mL) | (µg/mL) | (µg/mL) | (µg/mL) | (µg/mL) | (µg/mL) | (µg/mL) | - |
| **myristic acid (14:0)** | **1** | 44.18 | 39.78 | 23.09 | 8.43 | 192.15 | 28.66 | 52.84 | 212 | 89.21 | 79.28 | 49.76 | 12.95 | 282.43 | 52.27 | 112.60 | 203 |
| **pentadecanoic acid (15:0)** | **2** | 3.32 | 2.94 | 1.78 | 0.89 | 10.69 | 2.23 | 3.73 | 212 | 8.17 | 7.21 | 5.11 | 1.06 | 42.74 | 4.95 | 9.76 | 203 |
| **palmitic acid (16:0)** | **3** | 678.90 | 652.65 | 176.40 | 263.63 | 1405.32 | 553.31 | 791.55 | 212 | 731.45 | 701.24 | 208.05 | 295.87 | 1498.20 | 594.82 | 838.28 | 203 |
| **stearic acid (18:0)** | **4** | 156.20 | 143.78 | 49.48 | 61.82 | 322.29 | 120.41 | 180.53 | 212 | 206.67 | 201.97 | 66.83 | 64.86 | 467.60 | 159.98 | 246.10 | 203 |
| **arachidic acid (20:0)** | **5** | 1.75 | 1.61 | 0.77 | 0.60 | 7.34 | 1.30 | 1.96 | 212 | 2.81 | 2.50 | 1.48 | 0.84 | 9.49 | 1.82 | 3.35 | 203 |
| **behenic acid (22:0)** | **6** | 2.08 | 1.93 | 0.79 | 0.76 | 6.34 | 1.59 | 2.42 | 212 | 2.83 | 2.52 | 1.38 | 0.95 | 9.05 | 1.91 | 3.42 | 203 |
| **lignoceric acid (24:0)** | **7** | 2.00 | 1.90 | 0.78 | 0.40 | 5.14 | 1.49 | 2.37 | 212 | 2.23 | 1.96 | 1.02 | 0.54 | 7.74 | 1.55 | 2.66 | 203 |
| **myristoleic acid (14:1n5)** | **8** | 3.01 | 2.34 | 2.57 | 0.36 | 23.44 | 1.48 | 3.56 | 212 | 5.26 | 4.19 | 4.32 | 0.39 | 31.76 | 2.45 | 6.38 | 203 |
| **palmitoleic acid (16:1n7)** | **9** | 73.77 | 67.47 | 38.87 | 13.73 | 245.66 | 45.23 | 89.83 | 212 | 51.62 | 44.61 | 32.26 | 9.96 | 209.22 | 28.03 | 67.48 | 203 |
| **vaccenic acid (18:1n7)** | **10** | 60.23 | 55.40 | 22.89 | 22.52 | 159.19 | 44.77 | 72.82 | 212 | 52.98 | 50.62 | 18.43 | 14.21 | 129.01 | 39.66 | 64.00 | 203 |
| **oleic acid (18:1n9)** | **11** | 633.80 | 597.70 | 217.40 | 205.92 | 1708.74 | 496.13 | 757.54 | 212 | 675.67 | 632.81 | 239.67 | 215.97 | 1441.10 | 513.12 | 800.51 | 203 |
| **cis-11-eicosaenoic acid (20:1n9)** | **12** | 3.83 | 3.44 | 1.61 | 1.12 | 13.55 | 2.75 | 4.46 | 212 | 4.35 | 3.93 | 1.83 | 1.43 | 13.81 | 3.03 | 4.99 | 203 |
| **mead acid (20:3n9)** | **13** | 4.79 | 2.76 | 7.06 | 0.02 | 60.93 | 1.78 | 4.26 | 212 | 2.64 | 1.78 | 2.77 | 0.01 | 20.78 | 1.13 | 2.95 | 200 |
| **erucic acid (22:1n9)** | **14** | 1.07 | 0.93 | 0.62 | 0.29 | 3.49 | 0.66 | 1.30 | 95 | 1.05 | 0.87 | 0.67 | 0.22 | 3.89 | 0.65 | 1.24 | 91 |
| **nervonic acid (24:1n9)** | **15** | 5.65 | 5.36 | 2.12 | 1.85 | 14.22 | 4.00 | 6.92 | 212 | 4.32 | 3.97 | 1.71 | 2.06 | 14.62 | 3.08 | 5.14 | 202 |
| **linoleic acid (18:2n6)** | **16** | 669.59 | 655.88 | 255.35 | 70.72 | 1631.77 | 511.29 | 799.18 | 212 | 1089.29 | 1008.71 | 407.36 | 124.66 | 2586.14 | 824.64 | 1308.78 | 203 |
| **gamma-linolenic acid (18:3n6)** | **17** | 3.80 | 2.98 | 3.82 | 0.66 | 38.96 | 2.08 | 4.05 | 211 | 8.48 | 6.34 | 7.21 | 0.53 | 42.02 | 3.88 | 10.25 | 203 |
| **cis-11,14-eicosadienoic acid (20:2n6)** | **18** | 5.73 | 5.56 | 2.32 | 0.60 | 13.48 | 4.14 | 7.16 | 212 | 10.37 | 10.10 | 4.23 | 1.13 | 24.21 | 7.43 | 13.02 | 203 |
| **dihomo-gamma-linolenic acid (20:3n6)** | **19** | 23.21 | 21.09 | 10.23 | 4.63 | 69.57 | 16.08 | 28.67 | 212 | 31.86 | 30.60 | 13.84 | 6.14 | 88.74 | 21.47 | 39.69 | 203 |
| **arachidonic acid (20:4n6)** | **20** | 277.00 | 270.03 | 114.30 | 46.31 | 643.13 | 188.44 | 344.26 | 212 | 196.18 | 188.76 | 68.74 | 51.37 | 573.83 | 149.25 | 229.99 | 203 |
| **cis-13-16-docosadienoic acid (22:2n6)** | **21** | 1.91 | 1.87 | 0.63 | 0.88 | 3.79 | 1.51 | 2.09 | 19 | 1.45 | 1.46 | 0.33 | 0.66 | 1.87 | 1.25 | 1.73 | 15 |
| **adrenic acid (22:4n6)** | **22** | 1.06 | 1.06 | 0.48 | 0.02 | 3.00 | 0.73 | 1.34 | 210 | 1.01 | 0.96 | 0.48 | 0.04 | 3.06 | 0.70 | 1.28 | 202 |
| **osbond acid (22:5n6)** | **23** | 8.48 | 7.93 | 3.97 | 1.73 | 32.02 | 5.73 | 10.60 | 212 | 6.45 | 6.01 | 2.97 | 1.83 | 20.54 | 4.65 | 7.57 | 203 |
| **alpha-linolenic acid (18:3n3)** | **24** | 14.79 | 11.79 | 11.89 | 1.65 | 91.17 | 8.57 | 16.57 | 212 | 40.04 | 30.99 | 29.64 | 4.05 | 210.36 | 19.77 | 54.38 | 203 |
| **stearidonic acid (18:4n3)** | **25** | 0.66 | 0.35 | 0.88 | 0.14 | 3.63 | 0.25 | 0.54 | 15 | 0.76 | 0.61 | 0.53 | 0.23 | 3.26 | 0.42 | 0.93 | 88 |
| **eicosatetraenoic acid (20:4n3)** | **26** | 0.93 | 0.81 | 0.56 | 0.14 | 3.96 | 0.61 | 1.10 | 70 | 1.21 | 1.07 | 0.67 | 0.17 | 3.71 | 0.72 | 1.55 | 138 |
| **eicosapentaenoic acid (20:5n3)** | **27** | 8.90 | 7.33 | 7.32 | 0.63 | 78.95 | 4.60 | 10.80 | 212 | 6.61 | 5.65 | 3.86 | 1.11 | 22.09 | 4.00 | 8.29 | 202 |
| **docosapentaenoic acid (22:5n3)** | **28** | 10.70 | 10.31 | 4.64 | 1.68 | 42.62 | 7.47 | 12.82 | 212 | 9.99 | 9.45 | 3.93 | 1.46 | 34.32 | 7.27 | 11.94 | 203 |
| **docosahexaenoic acid (22:6n3)** | **29** | 137.55 | 128.96 | 80.27 | 9.52 | 320.00 | 74.30 | 173.76 | 212 | 91.12 | 81.27 | 46.52 | 13.92 | 320.00 | 59.19 | 114.17 | 203 |

**Supplemental Figure 1**. t-distributed stochastic neighbor embedding (t-SNE) of absolute concentrations performed on samples from both study timepoints (i.e., day 0 and day 10), plotting data points (each representing a sample) as scatter plots shown for patients ages 2 to 24 months (left column), 2 to <6 months (middle column), and 6 to 24 months (right column). Triangles indicate samples from emollient-treated and circles from no-emollient patients.


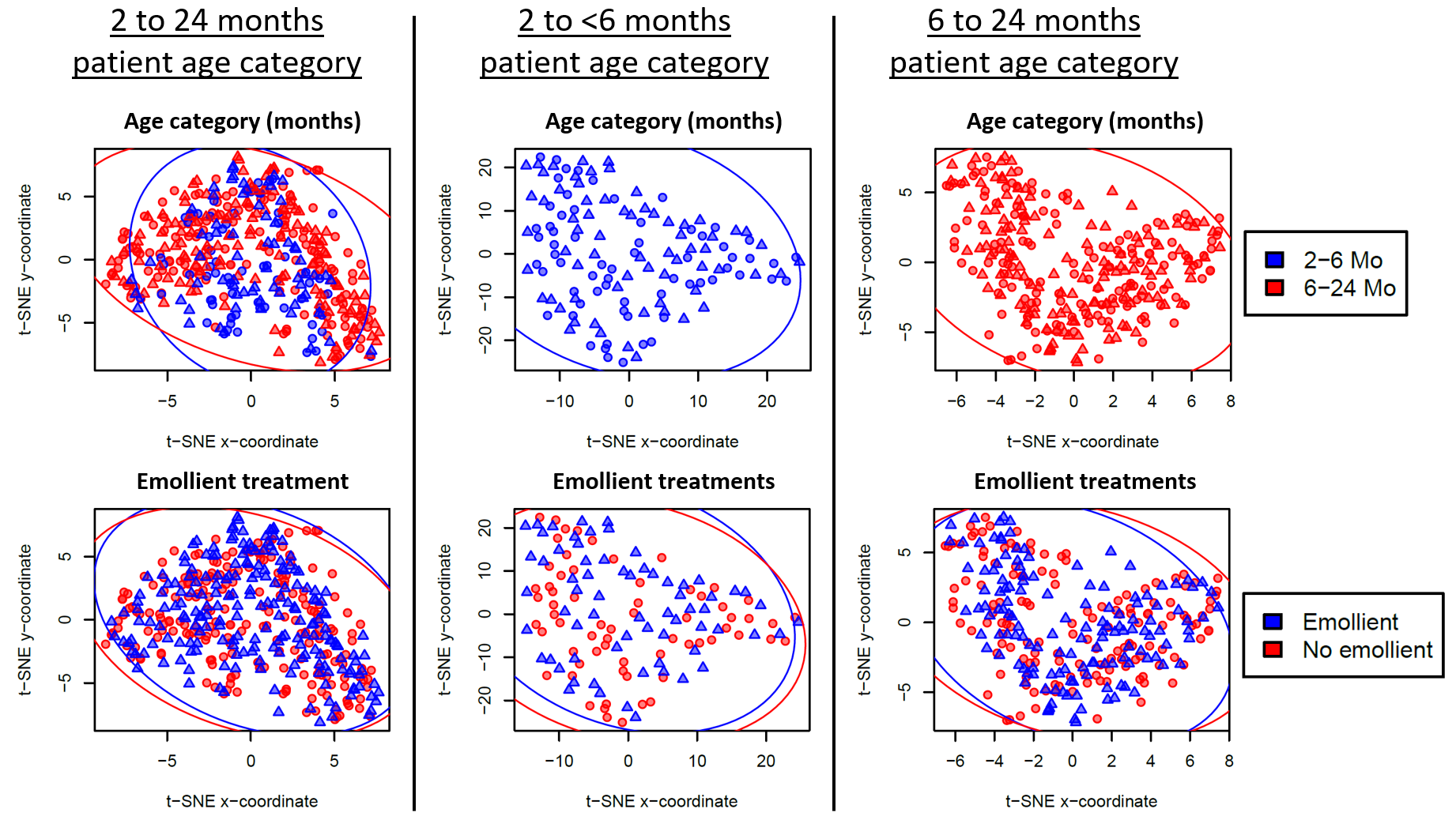


(Supplemental Figure 1, continued)


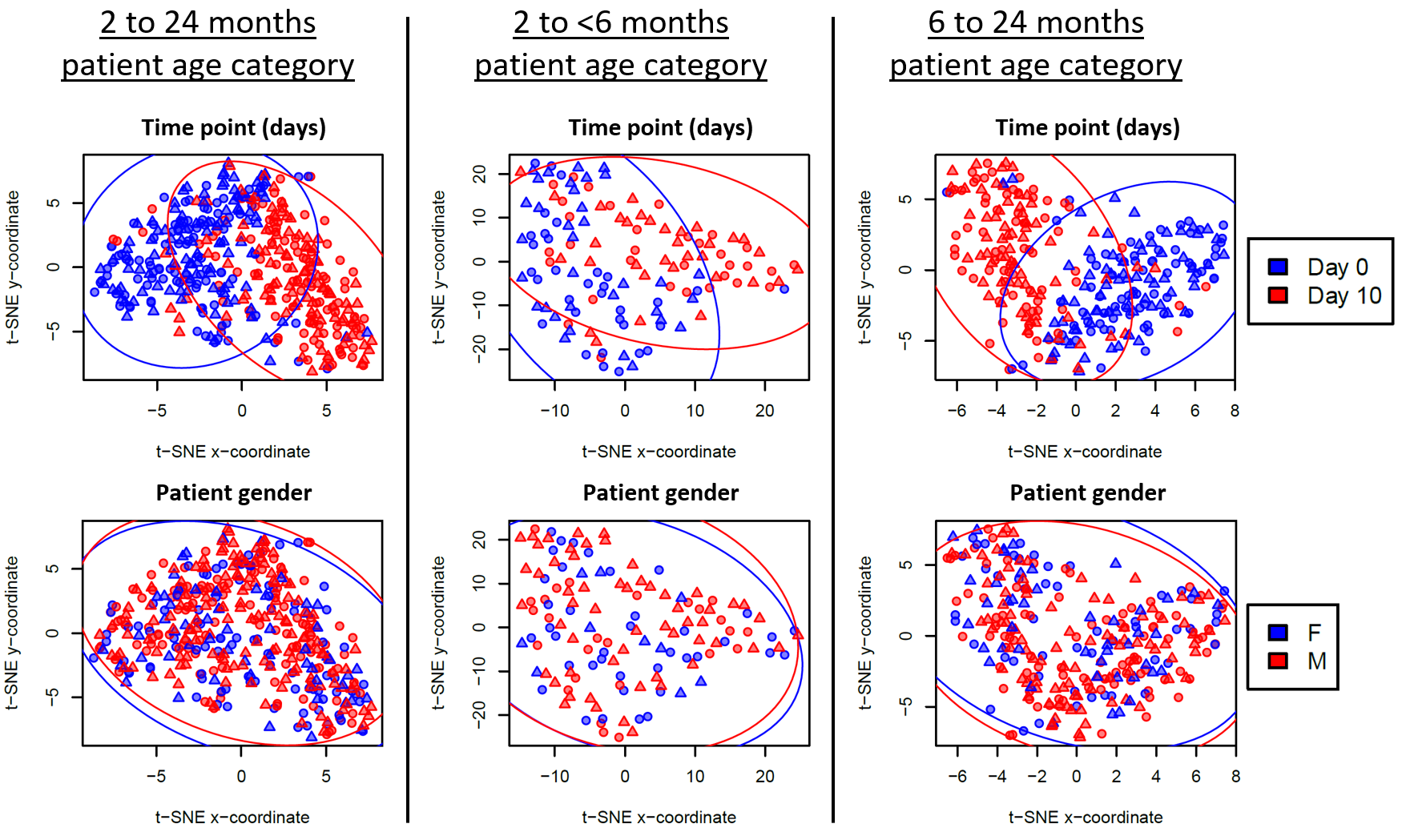


(Supplemental Figure 1, continued)


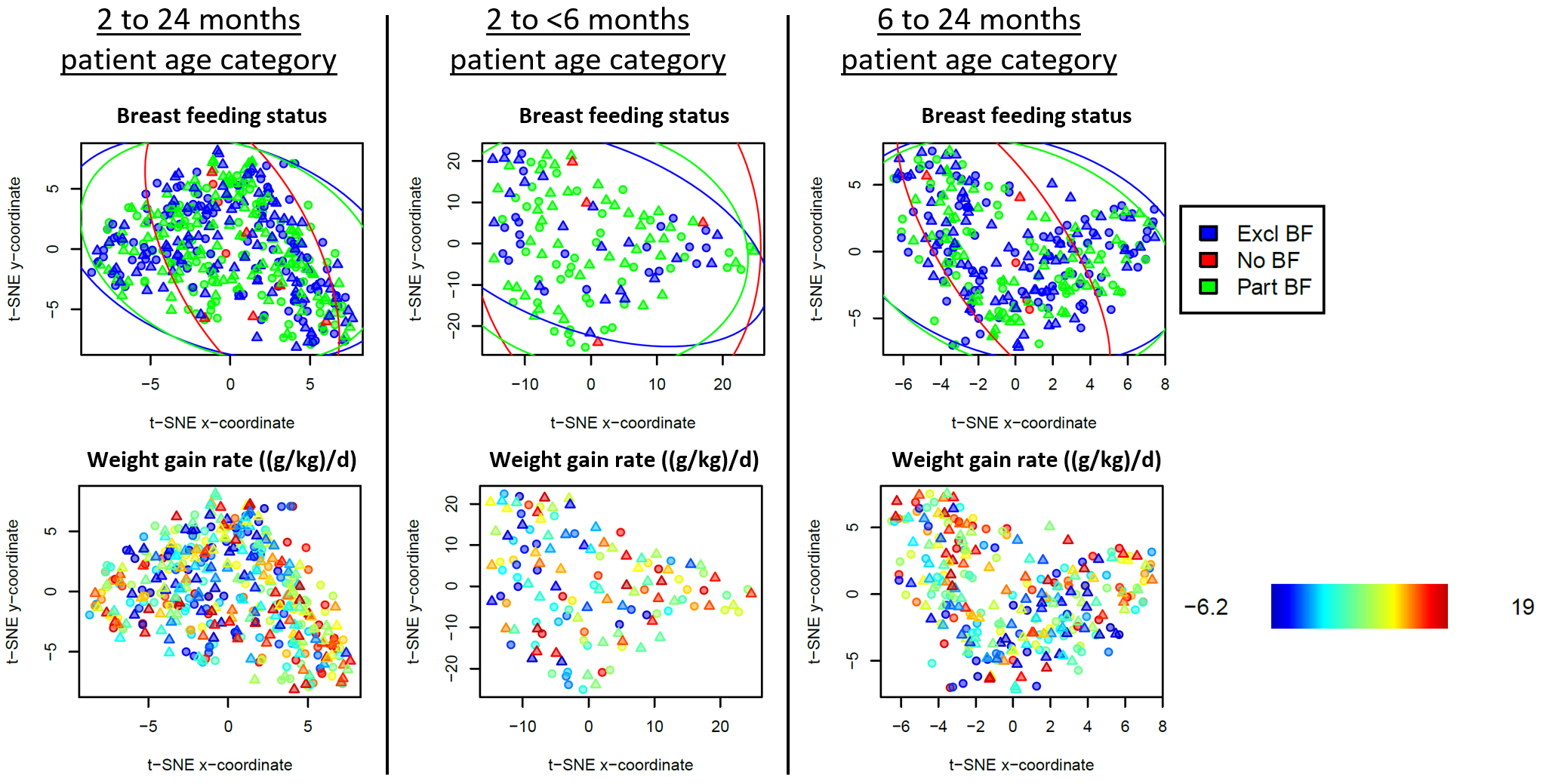


(Supplemental Figure 1, continued)


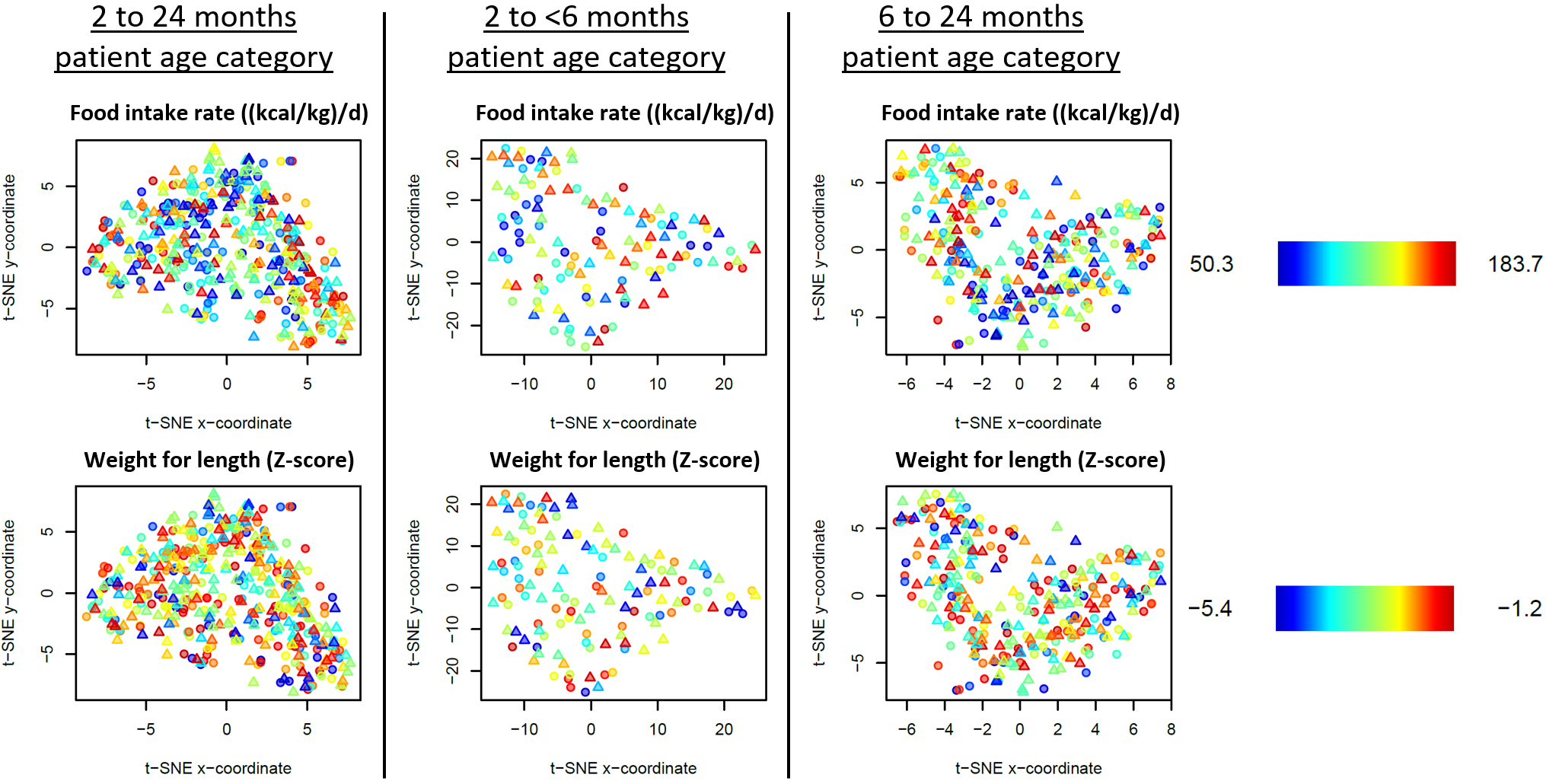


(Supplemental Figure 1, continued)


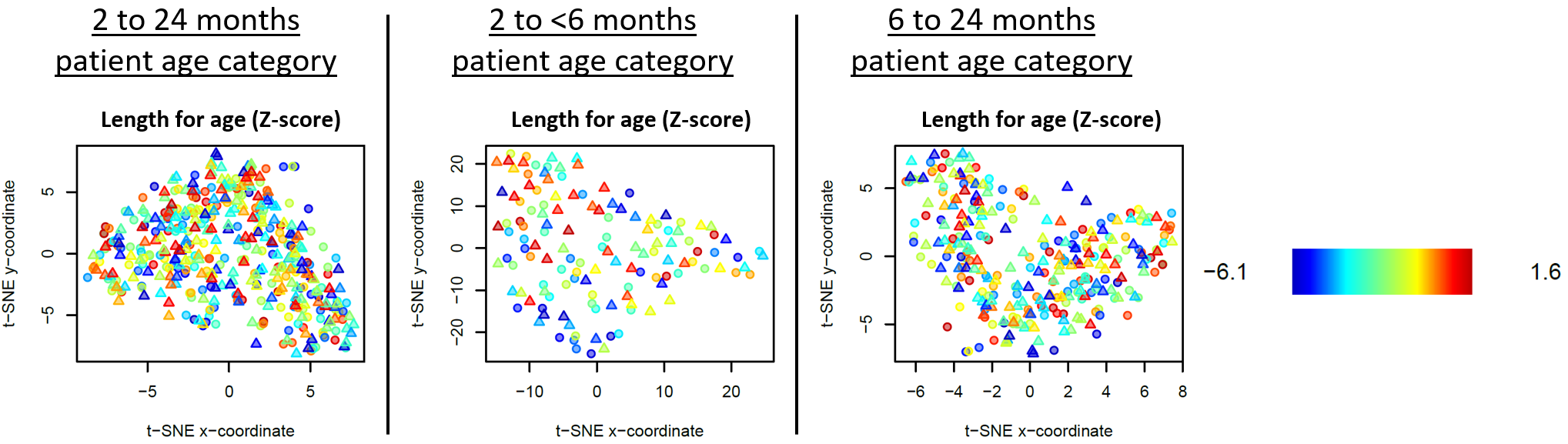

Supplement: Supplementary file 1 — Additional file 1: Supplemental Tables and figures. Supplemental Table 1. Composition of diets with calorie values for children with severe acute malnutrition (SAM) during rehabilitation. Supplemental Table 1A. Composition of liquid diets during acute phase of treatment of children with SAM. Supplemental Table 1B. Composition of semi-solid diets during rehabilitation phase of treatment for SAM. Supplemental Table 2. Specification of emollient (sunflower seed oil). Supplemental Table 3. 24-h Food Intake Chart. Supplemental Table 4. Lower and upper limits of detection provided by Metabolon for their free fatty acid analysis platform in ug/mL. Supplemental Table 5. Fatty acid measurements where an analyte was not detected. Suppplemental Table 6. Fatty acid measurements where an analyte was below the limit of quantitation (BLOQ). Supplemental Table 7. Fatty acid measurements where an analyte was above the limit of quantitation (ALOQ). Supplemental Table 8. Summary of out-of-range measurements of fatty acid levels. Supplemental Table 9. Metadata used for statistical analysis of fatty acid levels in children with severe acute malnutrition. Supplemental Table 10. Summary statistics on fatty acid concentrations. Supplemental Figure 1. t-distributed stochastic neighbor embedding (t-SNE) of absolute concentrations performed on samples from both study timepoints (i.e., day 0 and day 10), plotting data points (each representing a sample) as scatter plots shown for patients ages 2 to 24 months (left column), 2 to < 6 months (middle column), and 6 to 24 months (right column). Triangles indicate samples from emollient-treated and circles from no-emollient patients. [file 12937_2021_707_MOESM1_ESM.docx]
